# A Retrieved Context Model of Serial Recall and Free Recall

**DOI:** 10.1007/s42113-024-00221-9

**Published:** 2024-11-08

**Authors:** Lynn J. Lohnas

**Affiliations:** Department of Psychology, Syracuse University, 765 Irving Ave, Suite 352, Syracuse, NY 13210 USA

**Keywords:** Episodic memory, Free recall, Modeling, Retrieved context, Serial recall

## Abstract

A full characterization of memory must include how participants use exogenous and endogenous cues to guide retrieval. In free recall, in which endogenous cues play a large role, retrieved context theories have emerged as a leading explanation of data on the dynamics of memory search (Lohnas & Healey, *Psychology of Learning and Motivation*, *75*, 157–199, 2021). More recently, Logan and colleagues have advanced a retrieved context model to explain data on serial recall and motor production (Logan, *Psychological Review,* *125*(4), 453–485, 2018, *Psychological Review,* *128*(1), 1–44, 2021; Logan & Cox, *Psychological Review,* *128*(6), 1197–1205, 2021, *Psychological Review,* *130*(6), 1672–1687, 2023; Osth & Hurlstone, *Psychological Review*, *130*(2), 213–245, 2023). Comparisons of recall transitions have further highlighted similarities among these tasks (e.g., Bhatarah et al., *Memory & Cognition*, *36*(1), 20–34, 2008; Golomb et al., *Memory & Cognition*, *36*(5), 947–956, 2008). Here, I evaluate retrieved context theory’s ability to simultaneously account for data from these classic recall procedures. I show how a serial version of the context maintenance and retrieval model (termed sCMR) can account for dissociations between serial position curves and temporal clustering effects. I also show how sCMR can account for grouping effects using similar assumptions across recall procedures. The sCMR model provides a common theoretical framework to harmonize the disparate phenomena studied using these classic memory procedures, but also reveals the distinctions between serial and free recall through their relative dependence on different model-based mechanisms.

## Introduction

It is well established that memory performance varies with the type of retrieval cues provided at test (e.g., Greene, 1989; Tulving, 1985; Tulving & Pearlstone, 1966). However, in more open-ended recall tasks, it remains debated how participants use internally generated cues to retrieve information from memory. In recent decades, dozens of studies have attempted to resolve this debate with analyses in two classic recall paradigms: free recall and serial recall. In both tasks, participants study a sequence of individually presented items. In free recall, participants attempt to recall the items in the order they come to mind whereas in serial recall, participants attempt to recall the items in their presentation order. Each task gives rise to a characteristic serial position effect, with free recall exhibiting marked advantages in recall of recently studied items (the recency effect) and a smaller benefit for early list items (the primacy effect). Serial recall exhibits both effects as well, but has a much larger primacy effect than free recall and a small recency effect, often affecting only the last one or two items.

Based on these and other dissociations, distinct classes of theories have emerged to explain data from these two paradigms. Recent theories of free recall have emphasized the importance of contextual coding of memories as well as their semantic organization (Howard & Kahana, 2002a; Lohnas et al., 2015; Polyn et al., 2009; Sederberg et al., 2008). In contrast, theories of serial recall have emphasized positional coding, chunking, and the importance of tagging the beginning of the list to facilitate recall initiation (Brown et al., 2000; Burgess & Hitch, 2006; Farrell, 2012; Henson et al., 1996; Ladd & Woodworth, 1911; Lewandowsky & Farrell, 2008; Page & Norris, 1998). Although the development of these parallel theories proceeded for several decades, recent evidence suggests that the tasks exhibit some striking similarities, particularly in the manner in which participants transition among items (Bhatarah et al., 2006, 2008, 2009; Golomb et al., 2008; Grenfell-Essam & Ward, 2012; Grenfell-Essam et al., 2017; Spurgeon et al., 2014; Ward et al., 2010). These findings pave the way for a theoretical harmonization of the two procedures.

As one notable example, Farrell developed a chunking model to explain data from both free and serial recall, emphasizing that participants endogenously group each list into smaller groups (Farrell, 2012; Spurgeon et al., 2015). According to this model, each item is associated with a group context. Recall begins based on these group contexts to cue the context of a specific group. Recall then proceeds with the item associated with position 1 within that group, then the item associated with position 2, and so forth. This approach is similar to other serial recall models which use explicit positional codes to cue retrieval, but across all serial positions rather than within a group (e.g., Brown et al., 2000; Burgess & Hitch, 1999; Henson, 1998). The model of Farrell (2012) generalizes the use of groups and positional codes to both serial and free recall. Recall proceeds identically in the model in both recall tasks, except that the final group cues immediate free recall and the first group cues immediate serial recall. This model can account for recall initiation, recall transitions, recall accuracy, and recall errors across a variety of standard recall manipulations.

The current set of studies present an alternative model approach, showing how retrieved context theory can account for several major phenomena across the two paradigms. According to this theory, each studied item is associated with a temporal context state, and temporal context changes slowly with each studied item. Temporal context also serves as the retrieval cue during recall. The present model builds upon recent studies by Logan which show how principles of retrieved context theory offer a parsimonious account of several key features of data obtained in serial recall tasks (Logan, 2018, 2021; Logan & Cox, 2021, 2023, see also Osth & Hurlstone, 2023). Logan’s Context Retrieval and Updating (CRU) model extends principles of retrieved context theory from free recall (Howard & Kahana, 2002a; Polyn et al., 2009; Sederberg et al., 2008) such as the context maintenance and retrieval (CMR) model. Yet a unified retrieved context model across serial recall and free recall remains undeveloped.

**Fig. 1:**
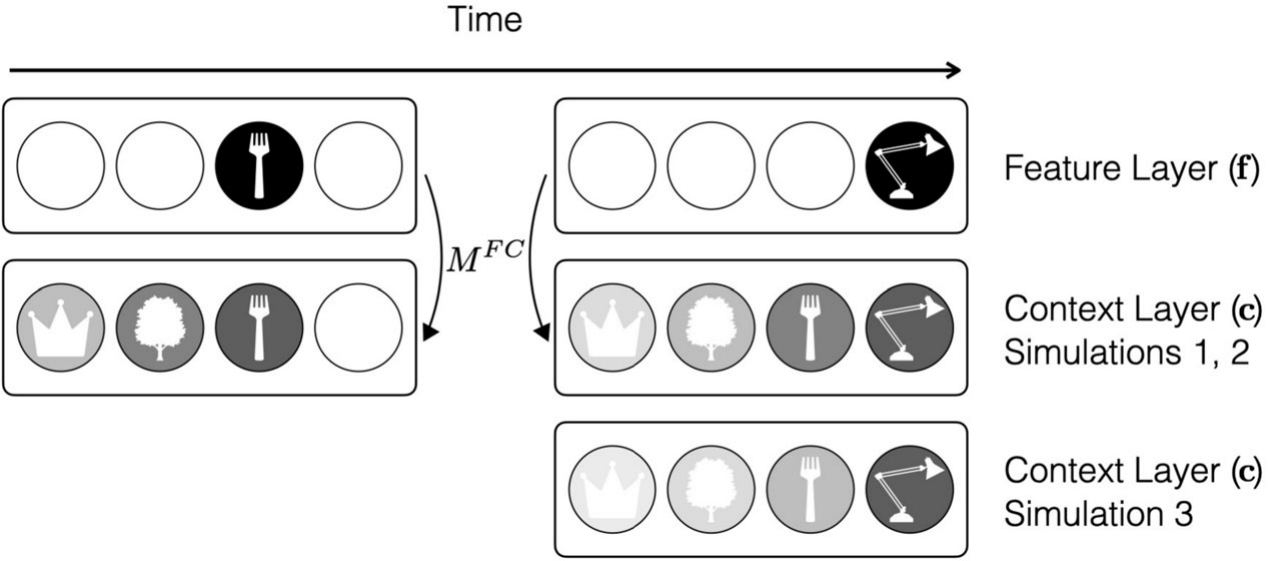
Encoding for the serial recall version of the context maintenance and retrieval model (sCMR). Each rounded rectangle represents a layer, actualized as a vector. Each circle within the rectangle represents an element of the vector. The background color of the elements, from black to white, corresponds to values ranging from one to zero, respectively. Left: fork was presented to the model most recently. Thus, in the feature layer, $\textbf{f}$, the element corresponding to the word fork is set to one, and all other elements are zero. This feature layer updates the context layer, $\textbf{c}$, through the association matrix ${\textbf {M}}^{FC}$. In the context layer, the element associated with fork has the greatest strength, indicated by the darkest shading of this element. However, previously presented words (crown, tree) also have nonzero strengths for their elements. As context updates, it downweighs prior context states, such that the context elements are a recency-weighted sum of previously presented items (i.e., the darkness of the elements is monotonically increasing with recently presented items). Right: The word lamp is next presented to the model after fork. Similar to the presentation of fork, in $\textbf{f}$, the feature element for the currently studied item is set to one and all other elements are zero. Also similar to the presentation of fork, the context layer $\textbf{c}$ is a recency-weighted sum of previously studied items. In Simulation 3 only, an additional temporal disruption item is presented between fork and lamp to indicate the start of a new group with lamp. As a result, the first three items are weighed more weakly in context (i.e., have lighter shading) than in Simulations 1 and 2. See text for further details

Importantly, although CRU makes accurate predictions in serial recall and CMR makes accurate predictions in free recall, these predictions have thus far been conducted more flexibly, with different models and some differing assumptions between paradigms. It may be that a model constricted to generally the same assumptions and parameters across tasks would be unable to account for findings from both tasks. In addition, to account for findings from serial recall, whole report and copy typing, CRU uses retrieval cues differently than retrieved context models of free recall. Thus, a more unified model approach helps to address the similarities and differences across tasks, especially with respect to the use of temporal context representations which have been found to be more critical in free recall than serial recall.

### Summary of Simulations

Here I evaluate predictions of the context maintenance and retrieval model generalized to serial recall (sCMR) in its ability to account for several key findings emerging from analyses of free and serial recall. In these simulations, sCMR changes two primary mechanisms to account for the differences between these recall tasks. First, sCMR changes the relative contributions of associations across tasks. When a participant recalls an item in free recall (e.g., fork in Fig. 1), this may remind them of other preceding items (e.g., tree) or of other semantic associates (e.g., spoon). However, in serial recall, the next correct recall should be lamp, and other associates would interfere with this correct recall. Thus, in serial recall, semantic associations and backward temporal associations contribute less to the context cue. As a second change, sCMR initiates recall differently across recall tasks. In free recall, sCMR accords with other retrieved context models and uses the current context to cue recall initiation. By contrast, in serial recall sCMR assumes that recall starts with the first studied item with high probability, aligned to the experiment data it is fitting. Models of serial recall often employ a similar mechanism, assuming a high probability to either recall the first item or to retrieve an effective cue for the first item (Anderson & Matessa, 1997; Brown et al., 2000; Burgess & Hitch, 1992; Caplan et al., 2022; Farrell, 2012; Henson, 1998; Lewandowsky & Farrell, 2008; Lewandowsky & Murdock, 1989; Page & Norris, 1998). With these proposed changes, I evaluate sCMR across a series of simulations.

In Simulation Set 1 I consider how sCMR can account for recall simultaneously in free recall and serial recall. I examine classic findings of recall probability and recall transitions in both paradigms, using a study in which the same set of participants performed one session of each recall task. To foreshadow the results, sCMR makes accurate predictions for most participants, and the distributions of model parameters across participants provide further insights into model mechanisms which vary across tasks.

In Simulation Set 2 I evaluate sCMR’s ability to capture recall transitions which have been argued as evidence against core assumptions included in retrieved context models (Farrell et al., 2013; Henson, 1996; Osth & Dennis, 2015a). Considerable prior research on free recall has highlighted the critical role that previously recalled items play in cuing subsequent responses (see Lohnas, in press, for a review). These studies have demonstrated that participants tend to transition between items studies in neighboring serial positions (henceforth, the *contiguity effect*; Healey et al., 2019; Kahana, 1996), and these transitions have a forward bias. Serial recall exhibits a similar pattern: following an omission, participants tend to make transitions to the prior or subsequent item, known as fill-in and in-fill errors, respectively (Farrell et al., 2013; Henson, 1996; Logan, 2021; Osth & Dennis, 2015a; Page & Norris, 1998; Solway et al., 2012). The nature of these transitions has recently emerged as a touchstone, distinguishing predictions of several models.

Positional code models predict that fill-in is more common than in-fill (Farrell, 2012; Henson, 1998; Henson et al., 1996; Page & Norris, 1998). When such a model is attempting to recall the *n*th item, broadly speaking the model assumes that early list items benefit from greater strength, so if the correct (*n*th) item is unavailable for recall, then the next most likely item to be recalled will be from an earlier list position (e.g., $n-1$). By contrast, retrieved context models more naturally predict in-fill. These models assume that recall of an item also updates context with information from that item. The updated context state promotes recall of items whose associated contexts include the just-recalled item, but items studied prior to the just-recalled item do not have that item in their context. Thus, items studied after the just-recalled item are more likely to be recalled next. For decades the fill-in effect has assumed to be evidence against models without positional codes such as retrieved context models.

However, Logan and Cox (2023) showed that CRU can predict the fill-in effect by assuming that recalling an error evokes retrieval of a start-of-list context. This promotes recall of items studied earlier in the list, including items studied before the just-recalled item. They noted however that this fit served as a qualitative proof of concept for retrieved context models and requires further characterization. Simulation Set 2 aims to use retrieved context models with more parsimonious principles from free recall, which generally do not use list context, to account for the fill-in effect. Taken together, the goal for sCMR is to account for both the in-fill effect, based on its natural tendencies, as well as the fill-in effect. To date, no model can account for all findings related to these transition errors.

Simulation Set 3 evaluates sCMR predictions of segmenting or chunking longer lists of serial recall into shorter groups. The presence of grouping has been a longstanding interest in serial recall (e.g., Farrell & Lewandowsky, 2004; Henson, 1999; Madigan, 1980; Maybery et al., 2002; Ryan, 1969; Wickelgren, 1967). Such a generalization is not trivial, because models which use a single temporal representation, like sCMR, have needed to incorporate positional codes to make accurate predictions of grouping (Liu & Caplan, 2020; Logan & Cox, 2023; Osth & Hurlstone, 2023). In addition, recently, there has been increasing interest at the intersection of segmenting continuous experience into meaningful events and the implications for episodic memory (for recent reviews, see Clewett et al., 2019; Radvansky & Zacks, 2017). In particular, grouping is posited to support within-group associations at the cost of across-group associations (e.g., Heusser et al., 2018; Lohnas et al., 2023; Radvansky & Copeland, 2006; Swallow et al., 2009; Zwaan, 1996). CMR could account for properties related to event segmentation and in free recall (Lohnas et al., 2023; Polyn et al., 2009), yet they posed more of a challenge for CRU in serial recall (Logan & Cox, 2023; Osth & Hurlstone, 2023). More broadly as well, the success of this set of simulations provides a quantitative application of assessing the role of temporal context representations in serial recall and free recall.

## Overview of sCMR: Context Maintenance and Retrieval Model of Episodic Recall

This section reviews principles of the retrieved context model framework, beginning with principles as they apply to the most common recall paradigm for this framework, free recall. We then turn to a description of the minimal changes from this free recall model in order to simulate serial recall. Retrieved context models in other paradigms also preserve these principles including final free recall (Howard et al., 2008), item recognition (Healey & Kahana, 2016; Rouhani et al., 2020) and cued recall (Howard et al., 2009; Zhou et al., 2023, see also Osth & Fox, 2019). Keeping the changes as minimal as possible serves to demonstrate the generalization of this class of models and also reveals the critical differences in model mechanisms across paradigms.

### Principles of the Context Maintenance and Retrieval Model

Figure 1 shows the basic structure of the model, which includes representations of items, contexts, and associations between them. Items are represented by the distribution of activation across elements (nodes) of a feature vector (layer), $\textbf{f}$. Each item is represented with a localist representation, using a vector of unit length with a single nonzero element. Each item has an associated context layer, represented by the distribution of activation across elements of a context vector $\textbf{c}$. These two vectors influence each other via associative matrices ${\textbf {M}}^{FC}$ and ${\textbf {M}}^{CF}$. The superscripts of these matrices indicate the direction of associations, e.g., ${\textbf {M}}^{FC}$ stores the strengths of associations from items to contexts. Each of these matrices is a weighted sum of a pre-experimental component and an experimental component. The pre-experimental component is fixed based on memories formed before the simulated experiment, and the experimental component is updated during the simulated experiment. Model parameters $\gamma _{FC},\gamma _{CF}$ scale the relative amount of experimental and pre-experimental contributions to the respective matrices ${\textbf {M}}^{FC},{\textbf {M}}^{CF}$.

Figure 1 shows the state of $\textbf{f}$ and $\textbf{c}$ after studying the third and fourth items in a sample list (fork, lamp). The associated item element is set to one in $\textbf{f}$, represented by the dark shading of that element only. The item then creates an input to context, $\textbf{c}^{\textbf{IN}}$ via the item-to-context association matrix:

1 $$\begin{aligned} \textbf{c}^{\textbf{IN}}_i = \textbf{M}^{FC} \textbf{f}_i. \end{aligned}$$

This input updates context according to the equation:

2 $$\begin{aligned} \textbf{c}_{i} = \rho _i \textbf{c}_{i-1} + \beta \textbf{c}^{\textbf{IN}}_i, \end{aligned}$$

where $\rho _i$ is set to normalize the input to context so that $||\textbf{c}_{i}||=1$:

3 $$\begin{aligned} \rho _i = \sqrt{1 + \beta ^2 [(\textbf{c}_{i-1} \cdot \textbf{c}^{\textbf{IN}}_i)^2 - 1]} - \beta (\textbf{c}_{i-1} \cdot \textbf{c}^{\textbf{IN}}_i). \end{aligned}$$

As each item updates context with Eq. 2, context becomes a recency-weighted sum of presented items. The context states in Fig. 1 also reflect this property, with the increasingly dark shading for increasingly recent items. The model parameter $\beta$ controls how much each studied item updates context, such that larger values of $\beta$ cause context states to change more with each context input. During encoding, context updates with the free parameter $\beta _{enc}$ for each studied item.

Each presented item is also associated with the previous state of context using a Hebbian learning rule, which updates the experimental components of each association matrix:

4 $$\begin{aligned} (\Delta {\textbf {M}}^{CF}_{exp})^\top = \Delta {\textbf {M}}^{FC}_{exp} = \textbf{c}_{i-1} \textbf{f}_{i}^\top \end{aligned}$$

In this way, sCMR associates an item with the temporal context in which it occurs. The associations in ${\textbf {M}}^{CF}$ are further scaled by a strength parameter which decreases with serial position, consistent with the notion that early list items benefit from increased encoding efficiency (Lohnas et al., 2020; Serruya et al., 2014; Tulving & Rosenbaum, 2006). Specifically, the item in serial position *i* updates ${\textbf {M}}^{CF}_{exp}$ according to the following:

5 $$\begin{aligned} \phi _i = \phi _s e^{-\phi _d (i - 1)} + 1, \end{aligned}$$

where $\phi _s$ scales the extra weight and $\phi _d$ scales the rate at which this advantage decays with serial position.

In free recall, the ${\textbf {M}}^{CF}$ matrix also encodes pre-experimental semantic associations among items, based on the hypothesis that similar items appear often in the same temporal contexts during one’s lifetime (Rao & Howard, 2008). The model parameter *s* scales the relative contribution of semantic versus experimental (episodic) associations to ${\textbf {M}}^{CF}$:

6 $$\begin{aligned} {\textbf {M}}^{CF} = (1-\gamma _{CF})(\textbf{I} + s{\textbf {M}}^{CF}_{pre}) + \gamma _{CF} \phi _i {\textbf {M}}^{CF}_{exp} \end{aligned}$$

where $\textbf{I}$ is an identity matrix.

Similarly, ${\textbf {M}}^{FC}_{exp}$ is a weighted sum of its pre-experimental and experimental components, the weighted tradeoff controlled by the $\gamma _{FC}$ parameter:

7 $$\begin{aligned} {\textbf {M}}^{FC} = (1 - \gamma _{FC}) {\textbf {M}}^{FC}_{pre} + \gamma _{FC} {\textbf {M}}^{FC}_{exp} \end{aligned}$$

where the pre-experimental component is defined as an identity matrix.

Once all items in a list have been presented, the recall period begins but how recall proceeds depends on the task. In free recall, the current state of context is used to cue recall of the first item. This context state assigns an activation for each item according to its associative strength to the current state of context:

8 $$\begin{aligned} \textbf{a} = {\textbf {M}}^{CF} \textbf{c}_i. \end{aligned}$$

where $\textbf{a}$ is a vector with each element corresponding to the activation of a studied item associated with the current context $\textbf{c}_i$. Because ${\textbf {M}}^{CF}$ is a weighted sum of temporal associations and semantic associations, with the latter scaled by the parameter *s* (see Eq. 6), larger values of *s* lead to larger contributions of semantic associations to the activation values. Further, the extra weighting of primacy items in ${\textbf {M}}^{CF}$ also leads to greater activation values for these items as well.

The activations are used as input to a Luce choice decision rule (Luce, 1959). By this rule, the probability that each item is recalled is related to its activation strength:

9 $$\begin{aligned} P(retrieve,i)= \frac{\textbf{a}_i^\tau }{\sum _k^l\textbf{a}_k^\tau } \end{aligned}$$

where $\tau$ is a model parameter. Larger values of $\tau$ magnify the differences between activation values of $\textbf{a}$, increasing larger values and decreasing smaller values. Thus, larger values of $\tau$ increase recall probability for items with greater activations, corresponding to items with greater associations to the current context. Regardless, items with greater activation values—whether due to stronger associations with temporal context-to-item associations, semantic context-to-item associations, or the increased weight due to the primacy scaling—are more likely to be recalled.

At each output position, the model may recall an item or may stop attempting recall for the list. The probability of stopping increases with output position *j* according to the following:

10 $$\begin{aligned} P(stop,j)=\theta _s e^{j\theta _r}. \end{aligned}$$

For both $\theta$ parameters, larger values increase the probability of stopping recall. Yet whereas $\theta _s$ scales the overall probability of stopping, such that larger values increase the probability of stopping across all output positions, $\theta _r$ scales the degree to which stopping increases with output position. Incorporating the probability of stopping with the probability of retrieval, the overall probability of retrieving item *i* at output position *j* is as follows:

11 $$\begin{aligned} P(i,j)= P(retrieve,i)(1-P(stop,j)). \end{aligned}$$

If the stop response is selected, then the recall period ends for the current list. The model’s memory is reset and then the presentation of the next list begins. If an item is recalled, it updates context using Eq. 2 with the free parameter $\beta _{rec}$. This updated context then leads to a new set of activations $\textbf{a}$, and the model attempts to recall another item. However, once an item is recalled, it cannot be recalled during the current study period. Although other retrieved context models invoke more realistic mechanisms for response suppression and response times (e.g., Duncan & Lewandowsky, 2005; Lohnas et al., 2015; Sederberg et al., 2008), the simplifying assumption of sCMR keeps the focus on the model’s predictions of correct recalls and order errors rather than on incorrect repetitions or response suppression.

### Model Modifications to Simulate Serial Recall

The model architecture and parameter values were identical across simulations of free recall and serial recall, with two exceptions described below.

#### Recall Initiation

In serial recall, participants are instructed to initiate recall with the first presented list item, and recall accuracy for this first item is relatively high. In many models of serial recall, it is assumed that participants can access the first item or its associated cue with high accuracy, due to greater attention, distinctiveness, and/or novelty experienced at the start of the list (e.g., Anderson & Matessa, 1997; Brown et al., 2000; Burgess & Hitch, 1992; Caplan et al., 2022; Farrell, 2012; Henson, 1998; Lewandowsky & Farrell, 2008; Lewandowsky & Murdock, 1989; Page & Norris, 1998). Rather than attempt to simulate an additional attentional or retrieval process, sCMR initiates recall based on the empirical results from the data it is simulating. For all simulations except 3a, these probabilities were based on the normalized distribution of probability of first recall.^1^ For Simulation 3a, in which only the serial position curve was available, the probability of recalling an item at the remaining serial positions was set to a uniform value across the other serial positions such that the overall recall probability of initiating with a correct list item had a sum of one.

Recall of this first item shares similar properties to all other recalled items in sCMR. Specifically, recall of the first item elicits retrieval of its associated context from study, and this retrieved context serves as the input to then update context (Eq. 2). The updated context is used to cue recall of the second item. Recall of this second item and subsequent items, as well as recall termination, is identical to free recall.

#### Parameters Controlling Pre-experimental to Experimental Item-Context Associations

The distinguishing difference between serial recall and free recall, the requirement to recall items in their studied order, led to two differences in model parameters between serial recall and free recall. First, whereas in free recall, a just-recalled item tends to cue recall of subsequently presented items, in serial recall, an item should cue only the immediately subsequent item. Thus, the parameter controlling this cuing based on experimentally formed associations varied between free recall and serial recall ($\gamma _{FC}; Eq.$ 7). Second, whereas in free recall, participants tend to successively recall items with shared semantic information (e.g., Cofer et al., 1966; Healey et al., 2019; Howard & Kahana, 2002b; Pollio et al., 1969; Polyn et al., 2009), in serial recall, such semantic information does not benefit recalling items in serial order. Thus, the serial recall variant assumes that semantic associations do not contribute to memory retrieval processes (i.e., the parameter weighing the relative contribution of semantic to episodic associations, *s*, is set to zero; Eq. 6). This simplifying assumption is also consistent with other models of serial recall (Anderson & Matessa, 1997; Brown et al., 2007; Burgess & Hitch, 2006; Farrell, 2012; Henson, 1998). In the “Discussion” section, we address the possibility that in serial recall, semantic associations should be reduced rather than set to zero.

### Parameter-Fitting Technique

A genetic algorithm determined each best-fitting parameter set. This algorithm search minimized the sum of squared errors between sCMR predictions and experimental data. Generally, this includes all data plotted in the main text except recall probability of the first item in serial recall because the variance of this datapoint did not rely on model parameters. However, some other exceptions are noted in each simulation.

The algorithm first randomly selected a set of 10,000 values for each parameter, drawn from uniform distributions, for an initial generation of 10,000 parameter sets. Next, 20 generations of 1000 parameter sets were calculated, where the top 20% of all parameter sets from the previous generation were used as parents for the next generation, with a mutation rate of 10% of each parameter range. From these 20,000 parameter sets, the 100 parameter sets with the smallest sum of squared errors were rerun for ten sets of the experimental data. From these 100 parameter sets, the parameter set with the smallest fitness value was deemed the best-fit parameter set.

As described in more detail below, for Simulation Set 1, the algorithm determined a parameter set for each participant. All other simulations aimed to serve as a proof of concept that sCMR could capture the mean performance across participants, and thus, the algorithm determined a single parameter parameter set fit to mean participant data. Because in Simulation Set 1 each of the data sets forming the basis of the search was from a single participant, all simulations except the final 100 were rerun for three sets of the data. Similarly, for Simulation 3b, given the sparser data per participant, all simulations except the final 100 were rerun for five sets of the data. For the remaining simulations, each parameter set was evaluated once with the data set.

## Simulation Set 1: Shared Temporal Dynamics Between Serial Recall and Free Recall

This first simulation aims to present a retrieved context account of serial recall with minimal changes from free recall. To date, retrieved context theory has been tested separately in free recall or in serial recall, but both tasks have not been examined using a single, constrained model framework. This leaves open the possibility that retrieved context models may require more fundamental changes to mechanisms or parameters between tasks. Such a possibility would preclude a parsimonious theory of episodic memory within the retrieved context framework and would challenge how retrieve context models can account for the demonstrated consistencies between serial and free recall, even when the recall task is postcued (e.g., Bhatarah et al., 2008; Grenfell-Essam & Ward, 2012; Ward et al., 2010). If the model can account for findings from both tasks successfully, this would serve as more than a sum of its parts. The model would not only explain findings from each task, but also elucidate the minimal set of model mechanisms which change between tasks.

### Method

I assess whether sCMR, which embeds the CMR retrieved context model of free recall, can predict the salient features of serial recall using a data set of younger adults from Golomb et al. (2008), available from http://memory.psych.upenn.edu/Data_Archive#2008. In this study, each participant performed one session each of free recall and serial recall. Session order was counterbalanced across participants, and the two sessions occurred at least 1 week apart. Aside from the instruction of recall order, all other aspects of the study were identical between sessions.^2^ In brief, in both sessions, lists consisting of ten words were presented visually on a computer screen for 1 s, and participants said the words aloud as they were presented. Across lists, words were drawn randomly without replacement and lists were presented in blocks with varying interstimulus intervals of 800 ms, 1200 ms, and 2400 ms. After the final list item, viewing three asterisks and hearing a tone indicated that participants had up to 60 s for verbal recall. However, participants could end the recall period sooner with a keypress.

A genetic algorithm determined a set of best-fit parameters for each participant (see previous section). Eight parameters varied with the algorithm but remained constant between the two recall tasks. Two additional free parameters varied between tasks. Both of these variable parameters control the relative influence of pre-experimental to experimental item-context associations (*s*; $\gamma _{FC}$; see Table 1). The $\gamma _{FC}$ parameter varied freely within a predetermined range for each recall task. The *s* parameter also varied freely for free recall simulations, but was set to zero for serial recall simulations (see “Model Modifications to Simulate Serial Recall”). For the free recall simulations, semantic similarity values were determined using Latent Semantic Analysis (LSA; Landauer & Dumais, 1997).

**Table 1:** The values for Simulation Set 1 are the mean across all 30 participants, whereas the remaining values are the single parameter set used to fit the data averaged across participants

Parameter	Sim. Set 1	Sim. 2a	Sim. 2b	Sim. 3a	Sim. 3b
$\beta _{enc}$	0.581 (0.030)	0.577	0.818	0.124	0.704
$\beta _{rec}$	0.790 (0.037)	0.581	0.537	0.922	0.600
$\phi _s$	2.500 (0.248)	2.086	4.159	3.420	4.453
$\phi _d$	1.195 (0.096)	0.134	0.933	0.526	2.114
$\theta _s$	0.035 (0.004)	0.005	0.041	0.050	0.061
$\theta _r$	0.358 (0.037)	0.772	0.408	0.018	0.210
$\tau$	4.259 (0.407)	11.389	5.194	9.466	4.378
$\gamma _{CF}$	0.852 (0.021)	0.671	0.950	0.535	0.883
$\gamma _{FC}$	0.255 (0.026)	0.520	0.344	0.921	0.240
$\gamma _{FC}^{fr}$	0.304 (0.045)	−	−	−	0.258
*s*	1.310 (0.136)	−	−	−	2.104
$\beta _{group}$	−	−	−	0.826	0.919

For Simulation 2b, the value of $\phi _d$ is the mean across the three participant bins. For details of the use and meaning of each parameter, see “Overview of sCMR: Context Maintenance and Retrieval Model of EpisodicRecall”

Sim., simulation

**Fig. 2:**
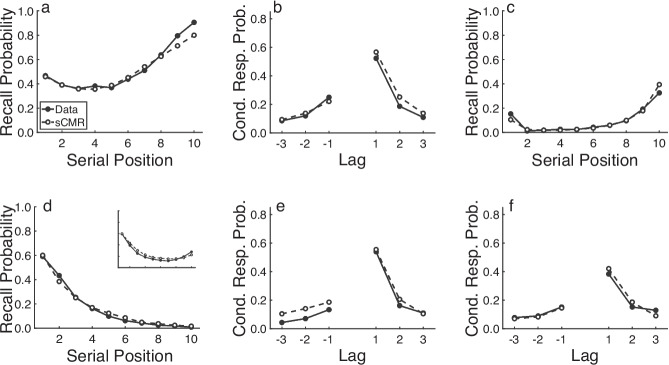
sCMR predictions and experimental data of the primacy, recency, and contiguity effects in free and serial recall. sCMR predicts accurately the plotted recall analyses. Top row: free recall. Bottom row: serial recall. **a**, **d** Serial position curves. Inset to **d**: Serial recall with relative order scoring (Solway et al., 2012). **b**, **e** Conditional response probability as a function of lag. **c** Probability of first recall. **f** Conditional response probability as a function of lag following the first-order error. Cond. Resp. Prob., conditional response probability. Data are from Golomb et al. (2008)

### Results

#### Free Recall

By starting with sCMR predictions of critical free recall phenomena, this provides an intuition of retrieved context models in their primary paradigm while ensuring that a simultaneous fit to serial recall does impair model predictions. Figure 2 presents sCMR predictions and empirical data averaged across participants (for individual participant fits, see Appendix, Figs. 9, 10, 11, 12, 13, and 14). Figure 2c shows that sCMR predicts participants’ tendency to initiate recall with the most recently presented items. In sCMR, the end-of-list context used to cue recall of the first item is a recency-weighted sum of presented items, and thus, more recently presented items dominate the retrieval cue (see Fig. 1). The stronger representation of recency items in the context cue also leads to sCMR’s prediction that recency items are more likely to be recalled across output positions (Fig. 2a). In free recall, the tendency to recall early list items, or the primacy effect, mainly reflects the increased context-item associations of early list items.

sCMR also predicts the temporal contiguity effect, a salient feature of free recall (Healey et al., 2019; Kahana, 1996). This is shown in Fig. 2b, which plots the probability of recalling an item from serial position *i*+lag immediately following recall of item *i*, conditional on the availability of item *i*+lag as a valid recall (lag-CRP; Kahana, 1996). sCMR naturally predicts the temporal contiguity effect because recall of an item leads to retrieval of its context, and this retrieved context is incorporated into the context used to cue recall of the next item. This updated cue promotes recall of items with shared temporal contexts of the just-recalled item. sCMR also predicts the forward asymmetry effect or the increased probability of forward over backward transitions (Kahana, 1996). When sCMR retrieves an item from memory, the item creates an input to context. This input is a weighted sum of its pre-experimental and experimental contexts (Eqs. 1, 7), and thus, both of these contexts contribute to the context state used to cue the next recall (Eq. 2). Importantly, an item’s pre-experimental context is only present in a temporal context after the item is studied. Thus, the pre-experimental context promotes recall of items studied after, but not before, the just-recalled item. Increasing the relative weight of this pre-experimental context, with free parameter $\gamma _{FC}$, therefore increases the forward asymmetry in the lag-CRP.

Taken together, sCMR accounts for classic recall dynamics in free recall. On the one hand, retrieved context models were developed for free recall, and several sets of model simulations have predicted these results previously (e.g., Sederberg et al., 2008). On the other hand, because sCMR had to make these predictions while also being constrained to making predictions of serial recall, it was not trivial that the model could maintain such accurate predictions. With this foundation, that sCMR meets the bar of previous retrieved context models of free recall, we now turn to its predictions in the newly applied paradigm of serial recall.

#### Serial Recall

Because sCMR assumes high accuracy for recalling the first list item, this promotes recall of items with similar temporal contexts also presented at the beginning of the list, and thus discourages recall of recency items. Along with the primacy gradient, these mechanisms allow for sCMR to account for the primacy effect in the serial position curve (Fig. 2d). When recall begins with an early list item, this promotes recall of items with similar temporal contexts, thus encouraging recall of other early list items and discouraging recall of recency items. As recall continues and context updates from early list items, context serves as a weaker cue for recency items. This contributes to reduced recall accuracy across output positions. For further contribution to this finding, with each output position, it is increasingly likely that the model will recall an incorrect item due to the noisiness of the retrieval process. This noise is independent across output positions, but increasing the number of noisy recalls increases the probability that at least one recall will be incorrect. As a final contributing factor to reduced recency in serial recall, the probability that the model stops recall increases with output position (see Eq. 10).

However, it is worth noting that many plotted serial position curves in serial recall studies do exhibit a weak recency effect. This recency effect may reflect one of two possibilities. First and most intuitively, participants may be more likely to recall items studied at the end of the list. We return to sCMR’s explanation of such a recency effect in Simulation 3a, where the serial position curve exhibits a recency effect. Second, the recency effect may emerge depending on the scoring method of the serial position curve. The present article uses an absolute scoring method, where an item studied in serial position *i* is scored as correct if it is recalled in output position *i*. However, serial recall experiments often report serial position curves using a relative order scoring metric, where an item is scored as correct when is correct relative to the previous item (for a comparison, see Dougherty et al., 2023). For instance, by one such metric, if a participant recalls successively the items studied in positions 1, 3, and 4, then item 4 would be considered correct because it follows item 3. With this more liberal scoring method, recency items are more likely to be scored as correct. Indeed, replotting the serial position curve using such a relative scoring method reveals a recency effect (Fig. 2d, inset). By this scoring metric, recall probability increases monotonically for the last three serial positions in both the experimental data and in sCMR’s predictions. For the main panel in that figure and more generally in this article, strict absolute scoring ensures that sCMR’s recall sequences align with the experimental data, as well as help to pinpoint potential sources of recall errors.

In the experimental data, the serial recall instruction leads to greater asymmetry in the lag-CRP than in free recall (Fig. 2e), which primarily reflects a reduction in $lag=-1$ transitions from free recall to serial recall reduced asymmetry (Ward et al., 2010, $M=0.250$ versus $M=0.133, SEM=0.0200, t(29)=5.83, p<.0001$). In sCMR, differences between tasks reflect the difference in parameters controlling the relative strength of pre-experimental to experimental item-to-context associations including $\gamma _{FC}$ (Eq. 7). This strength contributes to the role of experimental context in retrieval cues. When an item is recalled, its associated temporal context creates an input to the retrieval cue for the next recall (Eq. 2). As a result, stronger relative weighting of the experimental item-to-context associations causes more of an item’s experimental context to contribute to the recall cue. This experimental context encourages recall of items studied near the just-recalled item, whether occurring before or after on the list. However, in serial recall, it is not as beneficial to have items studied *before* the just-recalled item in the retrieval cue, because subsequent recalls should be from items studied *after* the just-recalled item. In contrast, in free recall, items studied before the just-recalled item can be correct recalls. Thus, the intuition is that, relative to free recall, $\gamma _{FC}$ values should be smaller in serial recall to downweigh the relative contribution of experimental item-to-context associations and reduce backward transitions. Across participants, there is a significant correlation between the reductions in $\gamma _{FC}$ values from free to serial recall with the reduction in $lag~=-1$ transitions from free to serial recall ($r=.81, p<.0001$). However, examining the distribution of $\gamma _{FC}$ values across participants, the mean is numerically but not statistically lower for serial recall than free recall ($\gamma _{FC}$ vs $\gamma ^{fr}_{FC}$, respectively, in Table 1; $p>.3$). Nonetheless, the significant correlation suggests that the $\gamma _{FC}$ parameter contributes to the change in temporal transitions across tasks even if the reduction in the parameter value is less reliable than the reduction in $lag~=-1$ transitions.

**Fig. 3:**
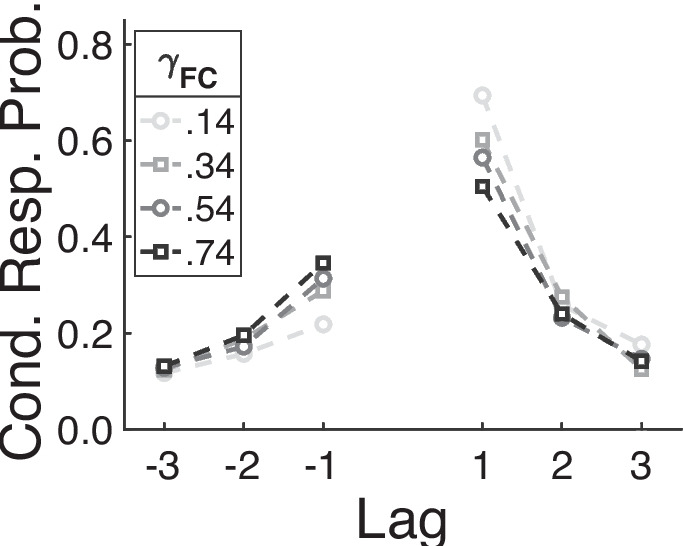
An intuition of the free model parameter $\gamma _{FC}$ with sCMR predictions of the lag-CRP in free recall across four values of $\gamma _{FC}$. All other values use the best-fit parameters from participant 26. Circles indicate values based on the algorithm search, with darker and lighter circles corresponding to the best-fit $\gamma _{FC}$ value from free recall and serial recall, respectively. Squares correspond to other intermediate values of $\gamma _{FC}$ for illustrative purposes. Data are simulated from Golomb et al. (2008)

As further intuition for the influence of $\gamma _{FC}$, Fig. 3 shows simulations in free recall with the best-fit parameter set from a sample participant and varying levels of $\gamma _{FC}$. In this figure, darker colors indicate greater values of $\gamma _{FC}$. As the value of $\gamma _{FC}$ increases, CRPs decrease at positive lags yet increase at negative lags, and thus reduces the forward asymmetry effect. The light gray circles correspond use the best-fit parameter values from free recall except using this participant’s best-fit $\gamma _{FC}$ value from serial recall. This lag-CRP, plotted with the smallest value of $\gamma _{FC}$, exhibits more forward asymmetry than the lag-CRP of the participant’s best-fit value of $\gamma _{FC}$ in free recall, plotted with dark gray circles.

In addition to changes in the $\gamma _{FC}$ parameter, differences in the *s* parameter also contribute to changes to temporal transitions in serial recall. In serial recall, *s* was set to zero, but in free recall, the distribution of *s* values was set on a closed interval containing zero, thus leading to significantly positive values ($M=1.31, SEM=0.136, t(29)=9.64, p<.0001$). Whereas the shared temporal context between studied items promotes successive recall of temporal neighbors, the shared semantic context between items can help to support recall of items with stronger semantic associations at all lags. Thus, an increase in *s* should increase reliance on semantic associations generally in conflict with temporal associations and the temporal contiguity effect. In each recall paradigm, I quantified each participant’s temporal contiguity effect with a temporal clustering score (Polyn et al., 2009). Across participants, the reduction in temporal clustering scores from serial recall to free recall correlated with their best-fit *s* parameters in free recall ($r=.63, p=.0002$), thus indicating the contribution of the *s* parameter to reduce the use of temporal associations. At the suggestion of a reviewer, I also calculated the correlation between participant’s best-fit *s* parameter and temporal clustering in serial recall, and the correlation was significant ($r=0.53, p=0.0028$). A participant who exhibits greater temporal clustering in serial recall may benefit more from using other forms of association when given the opportunity in free recall. By contrast, a participant who relies less on temporal cues in serial recall may more readily use the same cues in free recall.

As another approach to examine temporal organization in serial recall, the lag-CRP in Fig. 2 plots transitions to all items, and in serial recall, correct $lag=+1$ transitions dominate this function. The debate among theories of recall has centered around errors in recall transitions when the transition is an error and thus not $lag=+1$. Because items following an error may be clouded by the original error, recently, the convention is to calculate the lag-CRP from the first error on a list to another list item (Osth & Dennis, 2015a; Solway et al., 2012). Figure 2f plots this function, assuming that the preceding recalls are correct items and both the error and the following recall are neither repeats nor intrusions. In this study, participants exhibited an in-fill effect, with transitions more likely to $lag=+1$ than to $lag=-1$ ($M=0.383$ versus $0.152, SEM=0.0445, t(29)=5.20, p<.0001$). sCMR captures this effect for the same reason that it captures the forward asymmetry in the lag-CRP. In brief, recall of an error still retrieves its context, and this context updates the context cue for the next recall. The error’s pre-experimental context, now a part of the context cue, promotes recall of items studied after the error.

Taken together, sCMR can account for benchmark analyses of serial position effects and transitions in serial recall in the present data set. However, the in-fill effect is not always present in experimental data. Rather, sometimes participants demonstrate the fill-in effect, recalling the items prior to a recall error (Farrell et al., 2013; Henson, 1996; Logan, 2021; Osth & Dennis, 2015a). This effect has posed a challenge for retrieved context models such as sCMR, which traditionally in free recall predict a strong forward asymmetry. In the next simulation, we examine sCMR predictions of the fill-in effect.

## Simulation Set 2: A Retrieved Context Account of the Fill-In Effect

A special type of recall error has served as a defining distinction between serial recall theories. Specifically, when a participant mistakenly recalls an item too early (e.g., recalls item $i+1$ at output position *i*), theories are divided whether the participant next fills in with an earlier item such as *i* or in-fills with a later item such as $i+2$. Adding fuel to the theoretical debate, some serial recall studies exhibit the in-fill effect (e.g., Dougherty et al., 2023; Golomb et al., 2008; Solway et al., 2012) and some studies exhibit the fill-in effect (e.g., Farrell et al., 2013; Henson, 1996; Osth and Dennis, 2015a). Thus, although sCMR naturally explains the in-fill effect in Simulation Set 1, the aim of Simulation Set 2 is to examine sCMR’s ability to predict the fill-in effect. This aim is achieved through two sets of simulations using data from a study reported in Osth and Dennis (2015a), first demonstrating that sCMR can predict the fill-in effect and then exploring why sCMR can produce either the fill-in or the in-fill effect.

### Simulation 2a

This simulation aims to establish that sCMR can predict greater recall in the backward direction following a recall error. For this simulation, serving as a proof of concept, it is less important for sCMR to capture individual participant variability. Rather, it is more important for sCMR to simply predict a greater proportion of transitions for $lag=-1$ than $lag=+1$. Thus, Simulation 2a uses a single best-fit parameter set of sCMR which captures the pattern of transitions consistent with the fill-in effect, as well as the serial position curve.

**Fig. 4:**
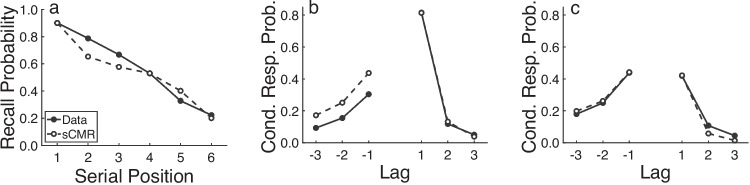
sCMR predictions and experimental data of the primacy, recency, and fill-in effects. sCMR provides an accurate account of the plotted serial recall analyses. **a** Serial position curves. **b** Conditional response probability as a function of lag. **c** Conditional response probability as a function of lag following the first-order error. Cond. Resp. Prob., conditional response probability. Data are from Osth and Dennis (2015a; b)

#### Method

I simulated sCMR using the list structure from a study presented in Osth and Dennis (2015a) using data available from https://osf.io/8zycm/. In this study, 100 participants performed serial recall of 62 lists each with six words. Each word was presented for 1 s followed by a blank interstimulus interval for 250 ms on a computer screen. Participants then viewed a prompt to type the studied words one at a time in order and had 20 s to do so. I chose to simulate the condition in which words were randomly selected without replacement across lists both because this design more closely parallels classic free recall studies and because sCMR’s memory resets between lists. In this condition, if participants knew that an item occurred at a specific position but could not remember the item, they could not provide a “pass” response but rather recalled the next word from the list. Again, this condition more closely matches free recall, in which task instructions usually do not require participants to specify if they know other items were studied in the list but cannot remember what they were. Further, because sCMR does not have a mechanism to enable a pass response this avoids having the present simulation obfuscate whether the model’s failure reflected incorrect assumptions of a pass mechanism or of retrieved context theory. It is worth noting that for this condition, Osth and Dennis (2015a) deemed that there was “neither a fill-in nor an in-fill effect” because the numerical advantage for transitions of $lag=-1$ over $lag=+1$ failed to reach significance across participants. For Simulation 2a, sCMR uses a single set of parameters to account for the mean values of this analysis without incorporating the variance. Thus, fitting sCMR the average data provides a meaningful standard for sCMR to capture an effect other than the in-fill effect, while simultaneously predicting recall probability and transitions across all output positions.

I used the same genetic algorithm procedure to determine the best-fit parameters as in Simulation Set 1. However, the algorithm only determined serial recall parameters, and thus, this model had nine parameters. The fitness of a parameter set incorporated all of the data plotted in Fig. 4, but to capture the qualitative pattern of the fill-in effect, the search also included the difference in the conditional response probabilities following the first order at *lag* = +1 and *lag* = -1. Further, because the aim was to capture the numerical advantage of backward over forward transitions as was present in the average data, the algorithm determined a single set of best-fit parameters based on this average data, rather than by individual participant.

#### Results

To lead with the critical result of this simulation, sCMR predicts the fill-in effect successfully, shown in Fig. 4c as greater conditional response probability of backward transitions than forward transitions following the first-order error. In sCMR, both the fill-in effect and the in-fill effect reflect the temporal contiguity effect. As explained in the previous section, sCMR naturally accounts for the contiguity effect because the just-recalled item reinstates its associated temporal context from the study phase and this updated context cues recall of items studied nearby in the list.

sCMR also accounts for the probability of recall by serial position (Fig. 4a) with recall more likely for early list items. As in Simulation Set 1, across output positions, the reduced recall advantage for later serial positions, increased probability of recall termination, and accumulation of errors lead to low recall of recency items. Thus, despite the similar serial position effects and correct transitions as in Simulation Set 1, in Simulation Set 2, sCMR can account for a greater probability of producing the fill-in effect.

Despite the backward asymmetry following a recall error, across all transitions, sCMR still predicts that forward transitions are more likely (Fig. 4b). This reflects both sCMR’s assumptions leading to the forward asymmetry effect and the inclusion of transitions from correct recalls. When the model has recalled items studied earlier in the list, these items no longer compete for recall and cannot be recalled again. Yet when the model skips an item and both of its neighbors are available for the next recall, backward transitions are more likely in this simulation. However, this still raises the question of why backward transitions are favored in this simulation, when sCMR generally favors forward transitions and predicted the in-fill effect in Simulation Set 1. The next simulation explores why sCMR can predict the fill-in effect in this simulation yet can predict the in-fill effect as well, by leveraging the variability in transitions across participants in this data set.

**Fig. 5:**
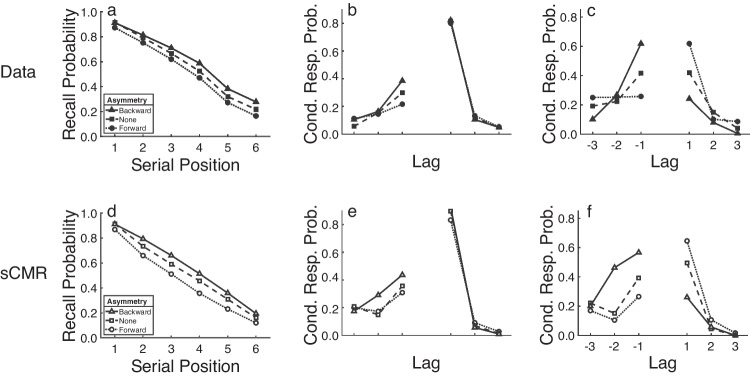
sCMR predictions and experimental data of the primacy, recency, and transition effects, when dividing participants into three bins based on their asymmetry in the conditional response probability as a function of lag (lag-CRP) following the first-order error. sCMR provides an accurate account of the plotted serial recall analyses. Top row: experimental data. Bottom row: sCMR predictions. **a**, **d** Serial position curves. **b**, **e** Conditional response probability as a function of lag. (Note the expanded *y*-axis due to sCMR’s prediction.) **c**, **f** Lag-CRP following the first-order error. Cond. Resp. Prob., conditional response probability. Data are from Osth and Dennis (2015a; b)

### Simulation 2b

Having established sCMR’s ability to capture the in-fill effect in Simulation Set 1 and the fill-in effect in Simulation 2a, we next ask how sCMR can explain both types of effects. We can explore this question using the same data set as Simulation 2a due to the across-participant variability in their lag-CRPs following the first-order error. To quantify this variability, we can define a participant’s asymmetry score this lag-CRP as their CRP value for lag=+1 minus their CRP value for lag=$-$1. Whereas positive asymmetry scores are consistent with the in-fill effect, negative asymmetry scores align with the fill-in effect. Participants were classified into three approximately equal bins based on their asymmetry scores (a positive score, a negative score, or a score close to zero; see “Method”). sCMR was then fit to these data with all but one parameter remaining constant between participant bins.

To motivate which parameter to vary, we consider a similar assumption to some serial recall models, that participants are more likely to make a transition of a negative lag due to the recall advantage for early list items (Henson, 1998; Page & Norris, 1998). In sCMR, greater recall of early list items reflects their stronger context-to-item associations. The first list item has the greatest strength, and this strength decreases with serial position. The free model parameter $\phi _d$ controls the degree to which strength decreases with serial position, and thus, this parameter serves as a natural one to vary between participant bins. Following this logic, a greater value of $\phi _d$ should lead to a faster decrease in recall across serial positions. Thus, recall of, and transitions to, early list items should be less likely, causing sCMR to predict the in-fill effect.

#### Method

I used the same data set as in Simulation 2a, but now subdivided the data into approximate terciles of participants based on their asymmetry scores, with scores greater than 0.1 ($N=33$), less than $-$0.1 ($N=37$), or an ambiguous effect ($N=30$). The threshold choice of 0.1 was meant to keep the same positive and negative threshold while dividing participants into approximate thirds and was made before running model simulations. Across these three terciles, the genetic algorithm procedure to determine the best-fit parameters was the same as Simulation 2a. However, just as in Simulation Set 1, where most parameters remained constant between serial recall and free recall; here, all parameters remained constant across participant terciles except $\phi _{d}$.

#### Results

The serial position curves provide an intuition of the influence of the $\phi _{d}$ parameter (Fig. 5b). Each line represents approximately a third of the participants. The circles represent the participants with the greatest reduction in the primacy effect, based both on the reduced recall probability and the highest value of $\phi _{d}$ (2.251). In Fig. 5f, this tercile exhibits the greatest in-fill effect. This greater value of $\phi _{d}$ reduces recall of early list items. Combined with sCMR’s natural tendency to predict the forward asymmetry effect, the weaker competition from early list items makes sCMR less likely to recall such items after the first error.

The participant tercile indicated with triangles exhibited the highest recall probabilities at earlier serial positions (Fig. 5a) and exhibited the fill-in effect (Fig. 5c). sCMR captures both of these effects by reducing the value of $\phi _{d}$ (0.346; Fig. 5b, f). Working through the outcome of this reduction, suppose sCMR studies the list in Fig. 1, then recalls crown,fork. As described in more detail in the free recall results of Simulation Set 1, the retrieved context from recalling fork should promote recall of subsequently studied items such as lamp. However, the strengthened context-to-item associations of tree will increase the probability of sCMR to recall an earlier list item like tree instead of lamp.

To summarize, among participant terciles, the highest value of $\phi _d$ leads to the in-fill effect and a lower value leads to the fill-in effect. It is perhaps less intuitive then that the lowest value of $\phi _d$ (0.202) produces a symmetric lag-CRP, arguably neither the fill-in nor the in-fill effect (plotted with squares in Fig. 5). As an intuition for this finding, suppose once again sCMR is tasked with which item to recall after recalling crown,fork, with both tree and lamp as possibilities. From sCMR’s ability to predict the fill-in effect, we know that a stronger primacy gradient can favor recall of the prior item tree. However, from sCMR’s ability to predict the in-fill effect, the forward asymmetric cues can favor recall of the next item lamp. When $\phi _d$ is sufficiently low, then the additional primacy strength does not decay as much from tree to lamp, and thus extends to lamp as well. Therefore, lamp is a stronger competitor to tree, and either item could be recalled with approximately equal probability. As further intuition for this point, decreasing $\phi _d$ further, to half of this best-fit value (0.101), leads sCMR to predict even less of a primacy effect (Fig. 15).

It is worth noting that other CMR papers generally assume that smaller values of $\phi _d$ produce a greater recall advantage for early list items (Polyn et al., 2009). However, critically, in those papers, CMR makes predictions of free recall. In that recall task, if fork cues strongly both lamp and tree, then both of these items could be recalled correctly. However, in serial recall, accuracy is greatest only when each recalled item promotes recall of the subsequently studied item in the list. Therefore, if fork cues strongly multiple primacy items for the next recall, rather than the next item alone, this can impair recall accuracy.

sCMR still can account for the lag-CRPs across all output positions (Fig. 5e). Notably, all participant terciles exhibited a strong tendency to make correct transitions of $lag=+1$ and differed in their CRPs of negative lag values. These lag-CRPs underscore the utility of examining the transition only after the first-order error (Solway et al., 2012), because when accuracy is high correct transitions tend to dominate the lag-CRP. Instead, the lag-CRPs following the first-order error reveal dissociations across participants and across model parameters.

## Simulation Set 3: Temporal Grouping Effects

This set of simulations examines the influence of imposing a grouping structure which encourages participants to group the longer sequence of the entire list into smaller subsequences or groups. Under such conditions, participants organize their recalls based on the temporal grouping structure. Notably, in serial recall, they exhibit primacy and recency effects within each group, mirroring the primacy and recency effect across the entire list (Farrell & Lewandowsky, 2004; Henson, 1996, 1999; Hitch et al., 1996; Madigan, 1980; Ryan, 1969). In serial recall, results from these studies have been interpreted as evidence against models without positional codes, and thus serve as a critical finding for sCMR to explain. Indeed, recent simulations of the CRU model, which shares retrieved context assumptions of sCMR, suggest that CRU requires positional codes to account for grouping effects in serial recall (Logan & Cox, 2023; Osth & Hurlstone, 2023).

Here, I examine recall grouping effects with studies using the common serial recall manipulation of having longer interstimulus intervals between groups (Farrell & Lewandowsky, 2004; Henson, 1996, 1999; Hitch et al., 1996; Madigan, 1980; Ryan, 1969; Spurgeon et al., 2015). In such experiments, participants study lists of items either as a single group or with a pause after every three items. When there is a temporal pause in the list, sCMR assumes that this induces a shift in temporal context, consistent with the mechanisms used in past retrieved context models (Logan & Cox, 2023; Lohnas et al., 2023; Osth & Hurlstone, 2023; Polyn et al., 2012, 2009). Practically, this disruption item updates context according to Eq. 1 with an additional free parameter ($\beta _{group}$). Otherwise, the model parameters remain constant between the grouped lists and ungrouped lists. This assumption not only constrains the model between list types, but also assesses whether retrieved context model assumptions of grouping in free recall generalize to serial recall. Although sCMR’s grouping assumption aligns with that of CMR for free recall (Lohnas et al., 2023; Polyn et al., 2012, 2009), past CMR simulations of grouping in free recall used groups with additional distinguishable features, such as different encoding tasks. Here, I examine whether sCMR can use the same temporal pause manipulation in serial recall to explain grouping effects when applied to free recall. Across tasks, sCMR only varies with the same two parameters as in Simulation Set 1.

This set of simulations is composed of two data sets. The first examines grouping effects using the same type of list structure simulated with the model of Farrell (2012) and CRU: serial recall of lists of nine items either studied as three groups of three or as a single long group. This serves to establish sCMR’s ability to account for typical grouping effects in serial recall. The second simulation examines sCMR’s ability to capture grouping effects in free recall and serial recall simultaneously with minimal changes across recall tasks and between grouped or ungrouped lists. The detailed recall sequences in the second simulation also provide insight into sCMR’s ability to account for positional errors, which have posed a challenge to retrieved context models.

### Simulation 3a

This simulation provides a proof of concept and intuition for sCMR to capture grouping effects in serial recall sing similar principles to other retrieved context models. To ensure sCMR captures all salient features, the data is fit to a meta-analysis which has canonical grouping effects and has been simulated with the Farrell (2012) model.

**Fig. 6:**
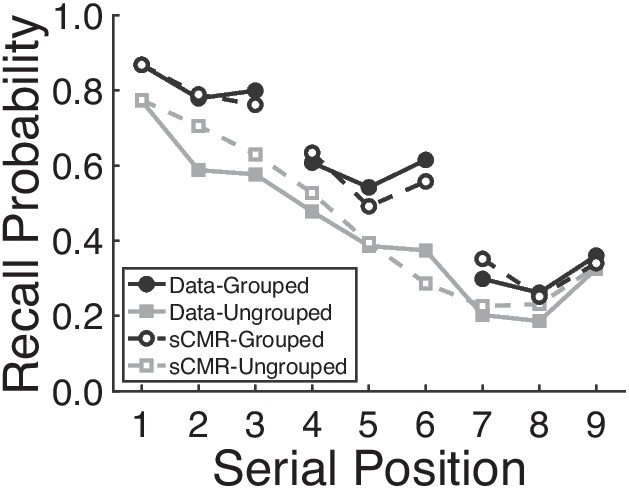
sCMR predictions of probability of recall as a function of serial position in grouped and ungrouped lists. In grouped lists, groups of three items were separated by a longer temporal pause before items 4 and 7, whereas ungrouped lists had consistent timing between all items. Serial position curves are extracted from Figure 4 of Farrell (2012), which was averaged across data sets using lists of digits from Henson (1996) and Hitch et al. (1996). sCMR accounts for the primary features of these serial position curves; see text for details

#### Method

I used the same genetic algorithm approach from Simulation Set 2 to fit sCMR to the serial position curves from Fig. 4 of Farrell (2012). The serial position curves present results averaged across several experiments in which participants studied lists of nine items as either three groups of three items, indicated by a longer temporal pause between groups, or as a single group (Henson, 1996; Hitch et al., 1996). Figure 4 from Farrell (2012) also presents model predictions of grouping in serial recall, and thus serves as a good comparison for sCMR. Aside from setting the parameter $\beta _{group}$ to 0 in the ungrouped lists and letting this parameter vary in the grouped lists, all other parameters remained constant between the grouped and ungrouped lists. For this mixture of experiments, I simulated an artificial data set of 50 “participants,” each of whom had 50 grouped lists and 50 ungrouped lists.

#### Results

Figure 6 shows that sCMR can account for the serial position curves with and without grouping. In the latter case, sCMR can account for the primacy and recency effects in a similar way to Simulation Set 1. Early list items benefit from the reinstatement of context from the first list item, and the additional associative strengths of primacy items to temporal context. The serial position curves in the plotted data sets (averaged from Henson, 1996; Hitch et al., 1996) exhibit a greater recency effect than in Simulation Set 1, but sCMR can account for this recency effect using the same mechanisms as in free recall.

The serial position curves between the ungrouped, control lists and the grouped lists differ at nearly all serial positions. If sCMR correctly recalls the first item then when compared to an ungrouped list, in a grouped list, the items studied after a pause are associated more weakly with the item’s reinstated context because the pause shifts temporal context further away from the context at the start of the list (see Fig. 1). As a result, the second and third items in the first group benefit from less competition from later list items, and these items boast greater recall probability in grouped than ungrouped lists.

Turning to recall of the fourth item in the list, recall of this item is more reduced from the preceding item in grouped than ungrouped lists. sCMR predicts this finding because recall of an item immediately prior to the pause will cue the next item more weakly than in an ungrouped list without a pause. This may also explain why items at the end of each group benefit from greater recall. In the ungrouped list, the greatest competition (i.e., next most likely recall) comes from the item studied after it, but in grouped lists, that next item (from the next group) is represented more weakly in context of the item which precedes it (in the current group). Thus, this across-group item serves as a weaker competitor during recall and increases the recall probability of end-of-group items. The reduction in competition from neighboring items in grouped lists may also explain why the model predicts accurately that recall is greater at each serial position in the grouped list compared to the ungrouped list. Further, this also helps to explain why the best-fit free parameter for the amount of context retrieval for each recalled item, $\beta _{rec}$, is higher for this simulation set (see Table 1); sCMR retrieves a larger range of temporal context states to ensure that the weakly represented item is still recalled next.

sCMR predicts a recency effect in both ungrouped and grouped lists. Items studied in later serial positions should be recalled in later output positions, and as output position increases, fewer items compete for recall in sCMR. This is due to sCMR’ assumption of absolute response suppression, or suppressing recall responses from already-recalled items. Thus, sCMR is less likely to confuse a recency item with other items. This is another common assumption in serial recall models (e.g., Brown et al., 2000; Farrell, 2012; Page & Norris, 1998). As further support for the role of response suppression here, CRU predicts less of a recency effect in grouped lists than sCMR, and CRU does not have an absolute suppression mechanism (Osth & Hurlstone, 2023). We return to the assumptions of this mechanism for sCMR in *Comparisons to other models of episodic recall*.

Having ensured sCMR’s ability to capture grouping effects in serial recall, we next examine how these principles can generalize across serial recall and free recall. Importantly, sCMR should maintain predictions of the salient features in these serial position curves while also capturing those of free recall.

### Simulation 3b

This simulation assesses sCMR’s ability to capture differences and similarities across recall tasks and across grouping conditions. Whereas in serial recall, grouping improves recall of the last item in each group, free recall does not tend to produce this advantage. More generally, whereas usually serial recall is greater for grouped than ungrouped lists (e.g., Simulation 3a), in free recall, the impact of grouping can be more subtle, with only the final group exhibiting greater recall (Gianutsos, 1972; Tzeng & Hung, 1973). Further, free recall of the first group can be greater for ungrouped than grouped lists (Gianutsos, 1972; Tzeng & Hung, 1973).

Although to date retrieved context models assume that a new group causes a disruption to temporal context, it would be ideal to assess this mechanism with as few sources of variation as possible between free recall and serial recall, in both experiment methods and sCMR mechanisms. In terms of experiment design, in comparison to the standard serial recall approach of temporal pauses, CMR simulations of free recall use different encoding tasks across groups (Lohnas et al., 2023; Polyn et al., 2012, 2009). Therefore, it is important to ascertain sCMR can capture grouping effects in a free recall task while more closely matching serial recall protocols, with as few changes to the model across recall tasks.

In addition, this simulation investigates participants’ error patterns in serial recall of grouped lists. In particular, participants are more likely to confuse positions across groups, mistakenly recalling the item studied in position *p* of group *g* instead in the position *p* but in a different group such as $g+1$ or $g-1$ (Brown et al., 2000; Farrell & Lewandowsky, 2004; Henson, 1996, 1999; Hitch et al., 1996; Hurlstone, 2019; Ng & Maybery, 2002; Ryan, 1969; Wickelgren, 1964). Importantly, this finding has been taken as evidence against models which conventionally do not contain positional codes, such as retrieved context models like sCMR. Instead, retrieved context models predict serial recall errors from items studied nearby in time to the just-recalled item (e.g., Fig. 2 e, f) or due to random noise. Indeed, CRU could capture interposition errors only with additional codes corresponding to the position within each group (Logan & Cox, 2023; Osth & Hurlstone, 2023). Similarly, Liu and Caplan (2020) developed a model in which, like sCMR and CRU, items are associated with a temporal representation, and temporal representations inform recall performance (Brown et al., 2007). This model also needed to include positional codes in order to capture primacy and recency effects within each group. At the same time, not all studies report findings consistent with interposition errors (Liu & Caplan, 2020; Shafaghat et al., 2024; Wickelgren, 1964). Nonetheless, to the extent that these effects are present in serial recall data, sCMR should be able to account for them.

This simulation uses data from Spurgeon et al. (2015), which manipulated recall task (free or serial) and grouping condition (grouped or ungrouped). Like the data in Simulation 3a, in the grouped condition, a temporal pause signified a new group after every three items. To account for grouping data across recall tasks, like Simulation Set 1, sCMR remains as consistent as possible and changes only two parameters ($\gamma _{FC}$ and *s*). Further, sCMR assumes that grouping evokes the same level of temporal disruption across recall tasks ($\beta _{group}$). If successful, sCMR should be able to still capture the serial position effects as in Simulation 3a, but also capture the less striking pattern of grouping in free recall.

#### Method

I assess whether sCMR can account for the salient features of a data set which manipulates both grouping and recall task (Spurgeon et al., 2015). In this study, there were 20 participants each in four conditions: grouped lists, serial recall; ungrouped lists, serial recall; grouped lists, free recall, ungrouped lists, free recall. This study also manipulated list-length within participants (ranging 1–12), and when studying each list participants did not know the list-length in advance. However, here, I simulate sCMR with the list-length = 8 condition, where grouping effects are more pronounced. Although Spurgeon et al. (2015) reported that the effects of grouping were not significant across serial positions, like Simulation Set 2, these still serve as a numerical benchmark for sCMR.

The experiment procedure was nearly identical across conditions. In brief, participants first viewed and heard a start marker, then after 1000 ms studied lists of words one at a time, with the word both presented visually on the screen and played auditorally. A 250-ms blank screen separated each word within a group in the grouped lists, as well as all items in ungrouped lists. In the grouped conditions, participants “were instructed to try to group in three’s” and a 1250 ms pause separated words across groups. Each word was studied for 750 ms in the ungrouped conditions, and the total presentation time of the list was matched in the grouped conditions. After the final list item, a grid appeared on the screen with as many rows as studied items. Participants had unlimited time to write their responses on a grid sheet one row at a time. Whereas in free recall they wrote their responses in successive grid rows, in serial recall, they could write their responses next to the corresponding position of the item. Although this allowed participants to skip over unknown items, sCMR does not have a way to omit items (see “Future Model Developments”). Therefore, sCMR simulations were compared to the absolute scoring of participants’ uninterrupted serial recall sequences. However, the general pattern of experiment results remains the same if scored based on grid position.

To fit sCMR to the list-length = 8 condition, the genetic algorithm searched for a single best-fit parameter set to account for the mean data across participants and conditions. Like Simulation Set 1, all parameters remained constant across recall tasks except *s* and $\gamma _{FC}$. Like Simulation 3a, $\beta _{group}$ was set to 0 in the ungrouped conditions and had a single nonzero value in the grouped conditions.

For the free recall simulations, semantic similarity values of studied words were determined using the SentenceTransformers Python library (Reimers & Gurevych, 2019). This library implements Sentence Bidirectional Encoder Representations from Transformer (SBERT), which formulates semantic similarity values based on the relationships and predictability of sentences and their components, including words. These values were chosen partially based on their availability, and partially because on standard benchmarks SBERT outperforms global vectors (GLoVe; Pennington et al., 2014; Reimers and Gurevych, 2019), a method which also relies on prediction-based semantic models yet outperforms LSA on semantic benchmarks including when incorporated into CMR (Morton & Polyn, 2016; Pennington et al., 2014). Nonetheless, the choice of semantic similarity structure is less critical for the present free recall simulations. Semantic similarity is not analyzed directly but rather its contributions serve as a source of recall organization secondary to contributions of presentation order, as is standard in lists composed of randomly selected, unrelated words (e.g., Healey & Uitvlugt, 2019; Polyn et al., 2011). Indeed, all of the major points described below for sCMR’s free recall predictions in Simulation 3b are maintained when using the parameters in Table 1 but with LSA values (data not shown).

**Fig. 7:**
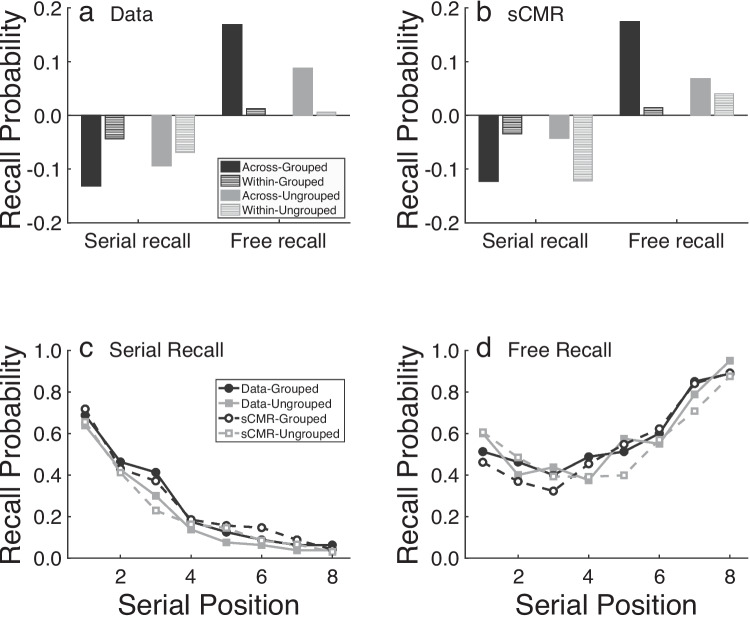
sCMR predictions of recall probability as a function of grouping (grouped lists or ungrouped lists) and recall task (free recall or serial recall). In grouped lists, groups of three items were separated by a longer temporal pause before items 4 and 7, whereas ungrouped lists had consistent timing between all items. **a**, **b** Mean differences in serial positions from the item at the end of the group, to its preceding temporal neighbor in the same group (within) or its subsequent temporal neighbor in a different group (across). **c**, **d** Serial position curves in serial recall (**c**) and free recall (**d**). Data are from Spurgeon et al. (2015)

**Fig. 8:**
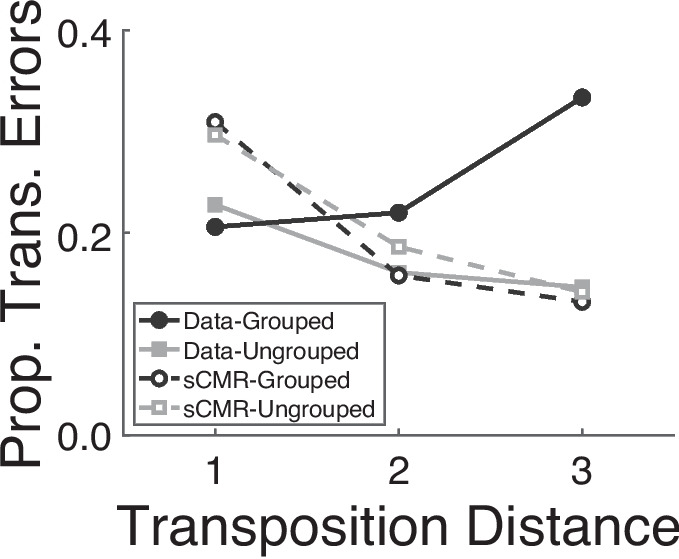
sCMR predictions of serial recall errors in grouped lists as a function of transposition distance. Prop. Trans., proportion of transitions. Data are from Spurgeon et al. (2015)

Due to the variability in the serial position curves, the genetic algorithm search determined the best-fit parameter values based on summary statistics of the serial position curve for items at the end of a group. In particular, these statistics were composed of the difference in serial position pairs from an item in group position 3 to group position 1 (averaged across serial position pairs 3,4 and 6,7), the difference in serial position pairs from an item in group position 2 to group position 3 (averaged across serial position pairs 2,3 and 5,6), and the mean recall probability across serial positions. These differences share similarities to the lag = +1 transitions calculated originally in Spurgeon et al. (2015), but sidestep the issues with grid order as well as that each recall task promotes transitions at different lags, and thus sCMR may still favor within group transitions without recalling neighboring items with lag = +1. The interposition analysis (Fig. 8) was also not included as part of the algorithm search, but rather included to demonstrate that sCMR cannot account for this effect simultaneously with the aforementioned serial position effects.

#### Results

Figure 7c shows the serial position curves across tasks. Like Simulation 3a, serial recall of grouped lists is generally better than ungrouped lists. Further, grouped lists boast elevated recall for items at the start and end of each group. Figure 7a summarizes the salient features of the serial position curves, based on the differences in recall probability across serial positions for items at the end of the group (3, 6). In particular, we can compare the recall probability of these items to their preceding neighbor (within group; 2, 5, respectively) and their following neighbor (across group; 4, 7, respectively). In this figure, bars indicate the recall probability of the later item minus the earlier item (e.g., recall probability at serial position 3 minus serial position 2). Thus, in serial recall negative values reflect the monotonically decreasing serial position curve. In grouped lists, there is a greater decrease in serial recall across groups versus within group, but matched positions in ungrouped lists do not exhibit this pattern as strikingly. sCMR predicts these effects because the retrieved context of a recalled item is a stronger cue for items within the same group than across groups (Fig. 7b). Thus, recall probability exhibits a greater reduction for the transitions across groups than within groups.

In addition to these salient features of serial recall, sCMR also accounts for critical grouping effects in free recall (Fig. 7d). First, in grouped lists, recall increases with group number and is particularly elevated for the final group. Based on sCMR’s assumption that the end of a group evokes a disruption to temporal context, items from earlier groups will be less similar to, and more weakly cued by, the current context. Because recall is competitive, this instead promotes recall of items from the final group. Following this logic, even the first group will suffer worse recall than the second group, because the temporal disruption item after the first group reduces the context shared between the context cue and the context associated with items from the first group.

sCMR’s predictions of increasing recall with group number in grouped lists can also be seen when compared to ungrouped lists. Specifically, there is reduced recall of items from the first group and elevated recall of items from the last group when compared to matched serial positions in the ungrouped lists. Although these interactions across grouping conditions are more subtle than the experimental data used here, other studies report significant differences between the first and last group across grouping conditions (Gianutsos, 1972). Nonetheless, sCMR’s predicted recall probabilities still align well with those from Spurgeon et al. (2015) and also importantly capture the differences in recall probability based on the preceding and following neighbors to an item at the end of a group (Fig. 7b). These differences are positive in free recall, reflecting that recall increases for items at the start of each group yet recall is not always elevated for the last item in each group.

The contrasting instructions between free and serial recall provide an intuition for sCMR’s predictions of the serial position differences in free recall. First, consider that in serial recall, the last item in a group (3,6) should follow recall of the middle group item (2,5, respectively). Items in the next group will not serve as strong competition to be recalled, and thus, recall of the final group item is more likely. However, in free recall, recall of the middle group item does not require recalling the next item in the sequence. Rather, sCMR may cue the next recall more strongly based on semantic associations (due to the increase in *s* from serial to free recall; see Table 1) and temporal associations other than at $lag=+1$ (due to the increase in $\gamma _{FC}$). Thus, in free recall, these additional associations may overshadow the shared temporal contexts of items studied in the same group.

Despite sCMR’s successes, it is also worth examining interposition errors, a grouping effect in serial recall which has challenged other retrieved context models (Logan & Cox, 2023; Osth & Hurlstone, 2023). If participants use positional codes within each group, then they may mistakenly recall an item at the correct position but in the wrong group (Brown et al., 2000; Farrell & Lewandowsky, 2004; Henson, 1996, 1999; Hitch et al., 1996; Hurlstone, 2019; Ng & Maybery, 2002; Ryan, 1969; Wickelgren, 1964). In the present study, with groups of length 3, participants should make more errors in grouped lists when the difference between an item’s serial position and output position, or transposition distance, is also 3. Matched serial positions in ungrouped lists, as well as transposition errors at other distances, can provide informative control conditions.

Figure 8 shows the proportion of errors as a function of transposition distance for grouped and ungrouped lists in serial recall. Errors at a transposition distance of 3 are more likely in grouped lists than in ungrouped lists, or even to the preceding distance of 2. Such results are consistent with a positional coding account. By contrast, sCMR predicts that transposition errors decrease with transposition distance. Similar to the intuition behind the temporal contiguity effect, each recalled item updates the context cue with its associated temporal context from the study phase, and this promotes recall of other items studied nearby in time.

Taken together, the temporal context representations in sCMR can account for some effects of grouping across both recall tasks. Importantly, sCMR assumes a temporal pause evokes the same change to temporal context across both tasks, and that model mechanisms are shared across tasks as in Simulation Set 1. At the same time, predictions of interposition suggest that participants may be using positional codes as well, a point elaborated in the “Discussion” section.

## Discussion

Theories of episodic memory aim to characterize how memories are represented as well as the mechanisms supporting successful encoding and retrieval. Much of the computational modeling work emerging from these theories has developed separately in free recall and in serial recall. Retrieved context models have had much success in accounting for recall dynamics in free recall (Howard & Kahana, 2002a; Lohnas & Healey, 2021; Lohnas et al., 2015; Polyn et al., 2009; Sederberg et al., 2008), and principles of these models have emerged as an important alternate account in serial recall (Logan, 2018, 2021; Logan & Cox, 2021, 2023; Osth & Hurlstone, 2023). Combined with recent empirical investigation (e.g., Bhatarah et al., 2008; Farrell et al., 2013; Osth & Dennis, 2015a, b; Solway et al., 2012), these lines of work underscore the parallels between, and the importance of assessing a generalized retrieve context model across, recall paradigms. Here, I examined three sets of simulations of sCMR, a generalized retrieved context model of free and serial recall. sCMR can account for several key findings using its core assumptions that each item is associated with a slowly changing temporal context state, temporal context is the recall cue, and recall of an item evokes retrieval of its associated context from the study phase.

Simulation Set 1 compares model predictions for participants who performed one session each of free recall and serial recall. sCMR makes accurate predictions of each participant’s recall patterns with changes to only two (out of ten) parameters, along with a difference in recall initiation, between recall sessions. sCMR accounts for the primacy effect in both tasks due to the stronger context-item strengths of early list items. sCMR accounts for a weaker recency effect in serial recall than free recall because the retrieved context of correctly recalled early list items drifts further from recency items, and with increasing output position recall errors and stopping are more likely. sCMR accounts for the temporal contiguity effect in both recall tasks because the temporal context of the just-recalled item most strongly contributes to the recall cue for the next item. Finally, sCMR accounts for reduced backward transitions in serial recall due to the two model parameters which varied between tasks; both of these parameters influence the contribution of experimental episodic associations to the recall cue. Thus, sCMR uses the same core principles, mechanisms, and representations for both recall paradigms but assumes that participants rely less on distant or backward associations in a task requiring recall in serial order.

Expanding on sCMR’s dissociations between recall para-digms, across tasks, sCMR uses different tradeoffs between pre-experimental and experimental contexts. Specifically, semantic context-to-item associations and backward episodic item-to-context associations contributed more strongly to the recall cue in free recall than in serial recall. This follows the intuition that using these two types of context information would be detrimental in serial recall, in which recall of an item should ideally cue the next studied item rather than a prior item or a semantic associate. Although sCMR updates the experimental item-context associations after studying an item, the relative weight of these experimental associations (versus pre-experimental associations) to the recall cue could be determined at the start of the recall period. In the present simulations, participants had a consistent recall task for an entire session or study, and thus, the parameter controlling the weights of experimental and pre-experimental associations was kept constant ($\gamma _{FC},\gamma _{CF},s$). However, if the type of recall test were postcued, then the model could adjust the association weights following the recall instructions.

With respect to the difference in recall initiation across tasks, in free recall, sCMR uses context to cue the first item, like most retrieved context models. However, in an immediate serial recall test, such a context cue will promote recall of recently studied items. sCMR assumes that recall initiation favors the first list item, like many models of serial recall (Anderson & Matessa, 1997; Brown et al., 2000; Burgess & Hitch, 1992; Farrell, 2012; Henson, 1998; Lewandowsky & Farrell, 2008; Lewandowsky & Murdock, 1989; Page & Norris, 1998). However, sCMR does not rely on context cues to recall this first item, but rather assumes context retrieval occurs automatically and consistently with participant data. We return to this simplifying assumption in “Future Model Developments.”

In Simulation Set 1, sCMR makes predictions consistent with the experimental data of Golomb et al. (2008), in which participants were more likely to make transitions in the forward direction in both free recall and serial recall. Yet in some serial recall studies, participants are more likely to fill in, or recall the preceding item, following a recall error of skipping an item. Simulation Set 2 thus examined sCMR’s ability to explain this finding. Because retrieved context models more naturally predict a forward asymmetry in free recall, the fill-in effect has been taken as evidence against models such as sCMR (Farrell et al., 2013; Henson, 1996; Logan, 2021; Osth & Dennis, 2015a). However, sCMR can account for the fill-in effect as well. Like the in-fill effect, sCMR predicts that the next recalled item, even if followed by the first-order error, is more likely to share temporal context with the current context state. Further, Simulation Set 2b demonstrated that sCMR accounts for tradeoffs between the fill-in versus in-fill effect based on a single parameter which controls the strength of the primacy effect. When early list items are more strongly associated with the context cue, this increases the probability that sCMR will recall the item preceding, rather than the item following, the recall error. Remarkably, some positional models use a similar mechanism, accounting for the fill-in effect based on stronger representations of early list items (e.g., Henson, 1998; Page & Norris, 1998). Further attesting that sCMR can account for this effect more naturally, with the same parameter set, the model also made accurate predictions of the serial position curve and lag-CRP including all output positions. Taken together, sCMR can account for both the fill-in effect and the in-fill effect, a flexibility which to date is less common in models of serial recall.

In Simulation Set 3, sCMR accounts for serial position curves of grouped and ungrouped lists with the same assumption as retrieved context models (Logan & Cox, 2023; Lohnas et al., 2023; Osth & Hurlstone, 2023; Polyn et al., 2012, 2009), that a new group induces a disruption to temporal context. Thus, across grouped and ungrouped lists, sCMR only needed to change the parameter controlling the degree of this grouping ($\beta _{group}$), but all other parameters remained constant. These disruption items lead to greater transitions between correct items in the same group but reduced transitions across groups, as well as primacy and recency effects within groups. This parallels the assumption for CMR2, a retrieved context model variant in which memories accumulate over multiple lists in a session: the model does not assume a separate list context, but a temporal disruption item was presented in between each study-recall cycle (Healey & Kahana, 2016; Lohnas et al., 2015; Pazdera & Kahana, 2023).

At the same time, with the temporal disruption grouping mechanism alone, sCMR cannot account for all of the grouping recall effects. Specifically, in Simulation 3b sCMR failed to account for the presence of interposition errors, the increased probability of recalling an item in the correct within-group position but across groups. To predict these errors, sCMR could take the same approach as CRU and incorporate positional codes (Logan & Cox, 2021; Osth & Hurlstone, 2023). However, future work remains to compare the advantages and disadvantages of positional code representations, whether these representations depend on temporal context (Logan & Cox, 2021) or are independent of context (e.g., Osth & Hurlstone, 2023). Because in serial recall positional intrusion errors serve as some of the strongest evidence for positional codes, empirical boundary conditions of positional errors will also inform future model development. For instance, some studies do not find significant interposition effects depending on the group size and recall order (Liu & Caplan, 2020; Shafaghat et al., 2024; Wickelgren, 1964). It will be important for future work to characterize these errors and thus the contributions of positional codes. As another example of the boundary conditions of positional codes, in free recall, few models incorporate positional codes. This has led to the success of retrieved context models without positional codes, including with grouping effects, as described further in the next section.

### Grouping and Segmentation in Retrieved Context Models

To date, retrieved context models have had success in accounting for grouping structures in free recall with experimental manipulations of grouping (Lohnas et al., 2023; Polyn et al., 2012, 2009). Without this grouping structure, the model does not assume that a longer list is divided into groups. However, there is empirical evidence that participants may acquire consistent grouping structures in recall tasks (e.g., Kahana & Jacobs, 2000; Madigan, 1980; Romani et al., 2016; Ryan, 1969; Wickelgren, 1967). On a theoretical level, Farrell’s (2012) model makes more accurate predictions in serial recall and free recall when assuming that participants automatically form groups from longer lists. For instance, the model can account for the nonmonotonicities in the serial position curve of the ungrouped lists from Fig. 6 when assuming that participants form groups in those lists.

Another potential approach to examine grouping, and indeed part of the motivation for Farrell’s model, comes from the event segmentation literature. Albeit gauged in different terminology than groups, this literature focuses on how people segment their continuous stream of experience into meaningful events (Radvansky & Zacks, 2014; Zacks et al., 2007). This literature also provides further foundation for sCMR’s assumption that a new group leads to a disruption to temporal context, even in situations where the new group (or event) is not a temporal pause. For instance, participants perceive two items (of matched temporal distance) as occurring further apart in time when they are from different events than when they are in the same event (Clewett et al., 2020; DuBrow & Davachi, 2013; Ezzyat & Davachi, 2014; Faber & Gennari, 2017; Lositsky et al., 2016). In addition, brain regions implicated in temporal information exhibit salient changes at event boundaries (e.g., Baldassano et al., 2017; Ezzyat and Davachi, 2014; Heusser et al., 2016; Lohnas et al., 2023; Lositsky et al., 2016).

Several computational models have been developed to explain interactions between episodic memory and grouping, through the lens of event segmentation. As one of the most relevant computational models to the present work, the Structured Event Memory model makes quantitative predictions of episodic memory and event structure based on predictability and similarity across events (Franklin et al., 2020). This model also makes accurate predictions of free recall and serial recall as a function of whether items were studied in the same or different events. Other event segmentation models, like sCMR, assume that temporal context changes slowly over time, and each item is associated with this slowly changing temporal context representation (Horner et al., 2016; Pu et al., 2022). Notably, the model of Pu et al. (2022) predicts a memory advantage for items studied earlier in an event, which also appears to be present in the serial position curves of the grouped data presented here. To date, these models cannot make predictions in recall tasks, but integrating their principles and testing their novel predictions with the sCMR framework provides another avenue to assess interactions between grouping and episodic recall. We next review other computational models which serve to generalize episodic memory across recall paradigms.

### Comparison to Other Models of Episodic Recall

The present set of simulation studies aims to provide a model of recall dynamics generalized across memory paradigms, and several other models share this goal. The Scale-Independent Memory, Perception, and Learning (SIMPLE) model (Brown et al., 2007) is one of the only other models that has been applied extensively to, and tested rigorously in, both recall tasks. Like sCMR, SIMPLE advocates for the importance of temporal representations. Specifically, SIMPLE assumes that each item’s memory representation is based on a logarithmic function of how recently the item was presented, and items with more distinctive representations boast greater recall probability. The logarithmic function leads to more distinct temporal representations of recency items, leading to the prediction of a recency effect in free recall. In serial recall, recency is reduced because more time has passed from the start of the recall period until the items should be recalled, not unlike the reduced recency in sCMR due to context drifting further away from these items. SIMPLE accounts for the primacy effect in both tasks with a less ad-hoc mechanism than sCMR. Items presented earlier in the list benefit from edge effects, as they have less temporal crowding from subsequently presented items. Although SIMPLE can account for transposition gradients in serial recall, to date, this model only produces recall probabilities for each presented item rather than recall dynamics.

Also like sCMR, SIMPLE can account for the serial position effects between grouped and ungrouped lists in serial recall and assumes that the start of each group is more temporally isolated from the prior item (Brown et al., 2007; Liu & Caplan, 2020). However, these temporal disruptions do not suffice on their own; SIMPLE needs to assume that each item is associated with a within-group position in order to capture the recall advantage for items at the start and end of each group, as well as for items studied in the first group. This reflects that this competition from items in different groups is too strong using temporal distinctiveness alone, and within-group positions help to reduce the competition. Using the example given in Brown et al. (2007), in lists with groups of three items, items 3 and 4 are close based on within-list temporal representations, but the completely different group positions promote the distinctiveness, and thus recall, of item 3. By contrast, because sCMR uses an evolving context to cue each recall, items studied in different groups are sufficiently weakly represented in this context cue. Thus, if the first two items were correctly recalled, the context cue would more weakly represent items from groups after the first group, such as item 4. Most likely, this suffices to reduce competition from item 4 and other temporal neighbors to item 3, promoting its recall. Although sCMR can capture recall probabilities of correct items without within-group representations, it currently cannot account for interposition errors (Simulation 3b). Future explorations of these models may be able to help capture why within-group position information is needed and is described further in “Future Model Developments.”

sCMR also shares conceptual similarities with models of free recall and serial recall which represent memories with item strength. Notably, the Adaptive Character of Thought-Rational (ACT-R) model (Anderson et al., 1998; Anderson & Matessa, 1997) and the Laminar Integrated Storage of Temporal Patterns for Associative Retrieval, Sequencing and Execution (LIST PARSE) model (Grossberg & Pearson, 2008) both assume that as each item is presented it enters a short-term memory buffer, and that memory strengths of presented items decay over time. Both of these models predict only recall probabilities, but share some similarities with sCMR. Both models assume that the recency effect in free recall reflects greater memory strength of recently studied items. After beginning recall with buffer items, ACT-R uses a set of strongly activated items to cue recall of other items. This is similar to sCMR’s recall cue with temporal context; both types of recall cues represent recently encountered items more strongly to promote the temporal contiguity effect. Further like sCMR, ACT-R can also assume a role for semantic information (c.f. Anderson & Bower, 1972).

In serial recall, the ACT-R model assumes that participants start at the beginning of the list and then recall proceeds in a forward direction. ACT-R predicts that recall probability is higher in earlier list positions because the model is more likely to make an error with each subsequent output position. In contrast, LIST PARSE predicts the primacy effect because items studied earlier in the list benefit from greater encoding strength, like sCMR’s primacy gradient. sCMR’s assumptions also parallel those of LIST PARSE with respect to grouping, as this latter model assumes temporal pauses impair integration between items. As a result, items in different groups are represented more distinctly and treated more like mini-lists with primacy and recency effects. In contrast, ACT-R can account for grouping effects with hierarchical representations and cuing similar to the Farrell (2012) model.

The Farrell (2012) model shares other similarities to sCMR as well. This model accounts for the primacy effect by assuming that there is a heightened probability for participants to initiate recall with the first list item, not unlike sCMR’s assumption for initiating serial recall. Similar to the ACT-R model, the model produces the recency effect in free recall because recall is more likely to initiate recall with the last group. The Farrell (2012) model also initiates recall with a group context, which promotes recall of other items in that same group. Although, unlike sCMR, the Farrell (2012) model always attempts to recall the item presented after the just-recalled item, this nonetheless leads to accurate predictions of the temporal contiguity and asymmetry effects in both recall tasks. In some cases, the Farrell (2012) model overpredicts the influence of grouping (Spurgeon et al., 2015), but this model was fit qualitatively rather than quantitatively.

With the models described thus far, sCMR shares assumptions with respect to mechanistic and representational differences across recall paradigms and with grouping. However, sCMR also helps to provide insight into the role of temporal context as a potential contribution to recall dynamics and recall accuracy. Similarly, the CRU model relies on principles of retrieved context theory to define and associate studied items with context states (Logan, 2018, 2021; Logan & Cox, 2021, 2023; Osth & Hurlstone, 2023). In particular, both models assume that each studied item is associated with a slowly changing context representation, and in serial recall, context serves as the retrieval cue. Both models also have had success in accounting for effects of serial position, recall transitions, and grouping. However, CRU has four noteworthy differences from sCMR, thus underscoring the importance of a unified retrieved context model of serial recall and free recall.

First, in CRU, the context retrieval cue updates with pre-experimental context only, whereas in sCMR, both pre-experimental and experimental contexts contribute. This assumption would be more challenging to bridge with retrieved contexts models of free recall or with several notable empirical findings in free recall which are more consistent specifically with the retrieval of experimental context (e.g., Manning et al., 2011; Talmi et al., 2019).

As a second difference between sCMR and CRU which dissociates their predictions, in the more recent version of CRU used to account for the fill-in effect (Logan & Cox, 2023), recalling an error in serial recall evokes retrieval of the context state from the start of the list. Whereas CRU simulates tasks requiring memory for serial order, retrieved context models of free recall assume that each retrieved item—error or not—evokes retrieval of its context. Thus, in contrast to CRU, sCMR does not retrieve context any differently following an order error in serial recall. However, because in shorter lists the context state will be closer to context from the start of the list after an order error, CRU and sCMR have a similar conceptual mechanism to support the fill-in effect: the context retrieval cue is more similar to items from the start of the list, thus supporting their recall over recently presented items.

As a third difference, CRU predicts less of a recency effect in grouped lists than sCMR (Osth & Hurlstone, 2023). Most likely, this reflects that sCMR assumes absolute response suppression: After sCMR recalls an item, the item is suppressed from being recalled again, or even from contributing to the decision rule (Equation 9; Brown et al., 2000; Farrell, 2012; Page & Norris, 1998). By contrast, CRU can recall an item more than once during a recall period (even though such repetitions are errors). With such an assumption, items in later serial positions, and thus generally later output positions, suffer from greater competition from other recalled items, which in turn reduces recall accuracy (Farrell & Lewandowsky, 2012). Although some retrieved context models have a response suppression mechanism (Lohnas et al., 2015), this relied on a different retrieval rule and would require further development to extend to sCMR. Empirically, future research should investigate how suppression varies across recall tasks as well.

As a fourth difference, sCMR assumes more idealized encoding and retrieval mechanisms than CRU. During encoding, sCMR stores the correct context and context-item associations for each item. These simplifying assumptions follow those of other models with idealized encoding processes (e.g., Brown et al., 2007; Henson, 1998; Page & Norris, 1998). By contrast, CRU can assume that there are errors in encoding (Logan, 2021; Osth & Hurlstone, 2023). Further, whereas sCMR always recalls the item retrieved from the Luce choice decision rule (Eq. 9), in some variants of CRU, each retrieved item then undergoes an output-stage confusion step (Logan, 2018; Osth & Hurlstone, 2023). Thus, CRU can have the correct item update context yet report the incorrect item. As Osth and Hurlstone (2023) assess, this is a necessary mechanism for CRU to account for patterns of recall in lists composed of some phonologically similar items (e.g., A, J, K) and some phonologically distinct items (e.g., B, H, X; Henson et al., 1996). Although sCMR has much success without assuming output-stage errors, sCMR would most likely have the same difficulties as CRU in attempting to capture phonological similarity effects without the output-stage error mechanism. This mechanism reflects another assumption typical for models of serial recall but not free recall (e.g., Henson, 1998; Page & Norris, 1998), and thus remains to be explored further in order to bridge retrieved context models across serial and free recall.

Whereas sCMR helps to shed light on shared representations, associations, and processes between free recall and serial recall, the differences between sCMR and CRU reveal some of sCMR’s remaining developments as a model of free recall extended to serial recall. We next review other model mechanisms and assumptions which may serve future advancements to sCMR.

### Future Model Developments

The present set of studies aims to extend the CMR model of free recall to serial recall, ensuring that the model as it currently stands can account for effects from both recall tasks. Thus, model development was intentionally limited to avoid the interpretation that the model must lean on new mechanisms in order to predict findings from free recall and serial recall simultaneously. However, this comes at the cost of sCMR making several simplifying assumptions. We review these below, as well as approaches to overcome these assumptions.

In serial recall, sCMR makes the simplifying assumption to initiate recall with the first list item on most trials. This assumption is similar conceptually to other models that, with high accuracy, recall begins with reinstatement of a cue from the beginning of the list (e.g., Anderson & Matessa, 1997; Brown et al., 2000; Burgess & Hitch, 1992; Farrell, 2012; Lehman & Malmberg, 2013; Lewandowsky & Murdock, 1989; Osth & Farrell, 2019). This assumption also sidesteps adjudicating between model failures of recall initiation versus model failures of retrieved context assumptions. However, as a result, sCMR cannot explain how participants initiate recall with the first list item. Specifically, sCMR cannot explain why participants can access the first list item with high but imperfect accuracy, nor can sCMR explain how context retrieval might differ for this first item.

sCMR may benefit from the assumption of other retrieved context models which assume an additional start item is encoded at the start of each list and can be reinstated at the start of the recall period (Healey & Wahlheim, 2023; Sederberg et al., 2011). Conceptually, this is similar to other models which assume highly accurate retrieval of a retrieval cue for the first item or of the first item itself (Anderson & Matessa, 1997; Brown et al., 2000; Burgess & Hitch, 1992; Caplan et al., 2022; Farrell, 2012; Henson, 1998; Lewandowsky & Farrell, 2008; Lewandowsky & Murdock, 1989; Page & Norris, 1998). Recently, Healey and Wahlheim (2023) showed that their model with this post-encoding pre-production reinstatement (PEPPR) mechanism accounts for patterns of free recall better than a variant which worked “backwards” to get to the beginning of the list or a prior list. sCMR may benefit from such a mechanism, and comparisons to other mechanisms would benefit from experiment designs in which recall initiation exhibits some variability, rather than nearly perfect as often occurs in serial recall of short lists (e.g., Simulation Set 2). However, future work remains to determine how serial recall evokes endogenous cues, including retrieving context, to ensure accurate recall initiation. In addition to how context retrieval takes place, it will also be important to discern how much context retrieval takes place. Whereas the PEPPR mechanism assumes that the start item retrieves context with a drift rate distinct from that of other recalled items, sCMR assumes that all endogenously retrieved items update context by the same amount ($\beta _{rec}$). If sCMR recalls the start or first item with a different context drift rate, this may also help to account for differences in the primacy effect across tasks. Currently, however, the simplified start mechanism suffices, as the primacy parameters ($\phi _s,\phi _d$) did not need to vary between serial recall and free recall in Simulation Sets 1 or 3. Yet given that recall initiation differs between serial recall and free recall, the simplistic initiation in serial recall should not take away from conclusions of the model’s generalization of memory processes across recall paradigms.

sCMR’s lack of positional codes also deserves further exploration, as this led to the failure to predict increased positional confusions in grouped lists of serial recall. Specifically, a recall error in a grouped list for the $n^{th}$ item from group *g* is more likely from the $n^{th}$ item of other groups (e.g., $g-1$,$g+1$; Brown et al., 2000; Farrell & Lewandowsky, 2004; Henson, 1996, 1999; Hitch et al., 1996; Hurlstone, 2019; Ng & Maybery, 2002; Ryan, 1969; Wickelgren, 1964). sCMR predicts that serial recall errors are generally from items with similar temporal context states, studied nearby item *n* (Fig. 8). Similarly, SIMPLE and CRU—other models founded on temporal representations—require positional codes to account for this effect (Liu & Caplan, 2020; Logan & Cox, 2023; Osth & Hurlstone, 2023). Thus, it seems reasonable to assume that sCMR will require positional codes as well, and remains a challenge for the present version of sCMR (Logan & Cox, 2023; Osth & Hurlstone, 2023). At the same time, not all studies exhibit interposition errors consistent with a pure positional coding account (Liu & Caplan, 2020; Shafaghat et al., 2024; Wickelgren, 1964), and thus, it will be important to characterize the boundary conditions of this effect. Further, it will also be important to explore the dependency between positional and temporal codes (Logan & Cox, 2021).

Fully developing a variant of sCMR with positional codes will also require the model to predict learning across lists instead of resetting memory between lists. This model could explore why items recalled (mistakenly) from prior lists are more likely to be recalled in the same serial or output position as their original lists, termed protrusions. Using the CRU model attempts to simulate this effect without adding positional codes have not been successful (Logan & Cox, 2023; Osth & Hurlstone, 2023). CRU’s predictions of protrusions were less inaccurate when it incorporated a start item, drawing on principles from a model which could use this start item and item-to-item associations to produce protrusions (Caplan et al., 2022). However, Logan and Cox (2023) interpreted their findings that CRU requires further development if only using a start item, and they instead favored a version of CRU which incorporated positional codes. Most likely, sCMR would face the same challenges of CRU rather than the success of the model of Caplan et al. (2022), and thus this remains part of the challenge of adding positional codes to sCMR. The sCMR model might also have difficult explaining other effects requiring learning by lists, which go against principles of sCMR. As one example, sCMR might also overpredict the recall advantage of keeping the relative order of list presentation consistent across lists while varying the positions (Ebenholtz, 1963; Kahana et al., 2010; Keppel, 1964; Winnick & Dornbush, 1963).

In addition to positional errors, sCMR also should be developed to explain semantic errors in serial recall. Presently, sCMR assumes, like many models, that semantic associations do not contribute to serial recall (Anderson & Matessa, 1997; Brown et al., 2007; Burgess & Hitch, 2006; Farrell, 2012; Henson, 1998). At the same time, there is some evidence that semantic similarity contributes to serial recall performance (e.g., Crowder, 1979; Golomb et al., 2008; Ishiguro & Saito, 2021; Kowialiewski et al., 2022; Poirier & Saint-Aubin, 1995). In the present introductory development of sCMR, I chose to prevent semantic associations from contributing to serial recall in order to isolate the contributions of temporal context. This clarifies sCMR’s predictive ability in relation to other models with similar assumptions. For instance, if sCMR had this additional degree of freedom in serial recall—to recall items based on their shared semantic similarity—this may aid sCMR in making backward transitions. Yet developing sCMR to explain semantic effects in serial recall would enhance understanding of the contributions of semantic associations across recall tasks. Further, if sCMR were developed to explain across-list effects, the model could be developed to explain the contribution of semantic similarity to recall errors from previous lists. In free recall, participants (and retrieved context models) produce this type of error due to their shared semantic similarity to current-list items (Healey & Kahana, 2016; Lohnas et al., 2015). How this extends to serial recall, whether with lists of unrelated words or contributions from other forms of longstanding knowledge, remains for future work.

As a final effect addressed in the present simulations but needing further development, we turn to the fill-in effect. By changing a single parameter in Simulation 2b, sCMR could account for either the in-fill effect or the fill-in effect. However, more work remains to be done for sCMR to capture the full scope of these transition effects. Because sCMR more naturally explains the in-fill effect, sCMR may not be able to predict studies with a very large fill-in effect, or the finding of a stable fill-in to in-fill ratio across output position in reconstruction of order (e.g., Surprenant et al., 2005). Further, sCMR, like many models of serial recall, also lacks the meta-cognitive machinery to explain why individual participants or different experiment conditions might produce variability in this effect.

There are several (nonexclusive) reasons why experiment manipulations may produce the fill-in effect or the in-fill effect (Farrell et al., 2013; Osth & Dennis, 2015a). One difference in task instructions is worth noting here because it requires a notable development to sCMR. This instruction can inflate the in-fill effect and occurs when a participant knows that the item they are about to next recall is not actually the next item in the sequence. For instance, suppose after a participant studies the list in Fig. 1, they recall crown. Although fork, lamp comes to mind, the participant knows that another item intervened between crown and fork, but they do not remember that it was tree. If all the participant can do is recall this next item, then fork would be recalled in output position 2. With an absolute scoring metric, fork is scored as the first-order error. This is indistinguishable from a participant who truly thought the sequence was crown,fork,lamp and produced an in-fill. The most straightforward way to distinguish between these possibilities is to instruct participants to report “blank” (e.g., crown,blank,fork,lamp). Because sCMR is more likely to produce the in-fill effect, it would be important to simulate serial recall with blanks included. However, presently, sCMR does not have a mechanism to omit items.

I propose one possible mechanism for sCMR to report omissions, based on the notion that if the item studied at serial position *i* was just recalled and its context retrieved, then the current context should be more similar to that of item $i+1$ than of $i+2$. With this assumption, we can use a similar mechanism to variants of CMR which compare the temporal context of a just-retrieved item to infer its location on a temporal timeline (Healey & Kahana, 2016; Lohnas et al., 2015). To illustrate, suppose sCMR recalls item *i*, and then item *j* wins the decision competition. Before recalling item *j*, sCMR would query the similarity between the current context and the input to context from the retrieval of item *j*. Measuring the similarity of these context vectors with a simple dot product yields a number between 0 and 1. If the similarity is below a threshold value, sCMR would report an omission and then recall item *j*. Otherwise, sCMR would recall item *j* as the next possible item. Of course, this mechanism should be tested and developed further, but provides one possible approach without introducing new model machinery.

Although sCMR may be able to account for the fill-in effect, making a single backward transition in error, sCMR may have more difficulty performing backward serial recall. In that task, participants must recall the studied items in their backward order, from the last item to the first. By dissociating serial position from output position, the backward recall task serves as an important benchmark and complements findings from standard forward serial recall (Anderson et al., 1998; Donolato et al., 2017; Liu & Caplan, 2020; Madigan, 1971). It may seem more challenging for sCMR to recall items in backward order, because core model assumptions tend to promote forward recall transitions. Although sCMR can favor backward transitions with the fill-in effect (Figs. 4c, 5f), this was for a special subset of items recalled mostly correctly in their forward order (Figs. 4b, 5e). However, backward recall could proceed as follows. If sCMR uses its current state of context to initiate recall as it does in free recall (e.g., Fig. 2c), recall should be more likely with an end-of-list item. Based on the temporal contiguity effect, recall of the nearest temporal neighbor should be more likely. By encouraging recall of temporal neighbors only, like in serial recall, then recall of one item should promote the next recall to be its not-yet-recalled, preceding temporal neighbor.

These potential challenges and developments do not need to make the sCMR framework obsolete. Rather, participants may rely on associative information in free recall and serial recall and additionally may rely on positional information in serial recall. However, the boundary conditions of using positional information remain to be characterized, especially in light of the current results. Further, Logan and Cox (2021, 2023) propose several mechanisms by which a retrieved context framework could incorporate positional coding when needed, while still maintaining associative information to support temporal contiguity effects. Future work remains to assess how temporal context works with or against positional information, but the current results reveal the importance of using model simulations for querying which effects retrieved context models can predict. Thus, the present work provides a piece to the puzzle by demonstrating that sCMR can account for findings from free serial and serial recall with minimal and informative changes between paradigms, including findings which have been taken as evidence against retrieved context theories.

## Conclusion

Episodic memory is generally assumed to reflect a common underlying set of cognitive representations and mechanisms. Yet memory performance varies with the memory task, partially due to differences in the presence and use of retrieval cues (e.g., Greene, 1989; Tulving, 1985; Tulving & Pearlstone, 1966). This has led to different theories for different memory paradigms, and in particular leaves more open questions for paradigms in which participants must generate their own internal cues. The present work aims to bridge the gap between two paradigms which require such internal cues, free recall and serial recall. sCMR, building on the retrieved context model framework in free recall, provides a quantitative framework and explanation for memory representations and retrieval mechanisms with minimal changes between paradigms. The results serve as more than a sum of their parts, providing a parsimonious account of these recall tasks while also highlighting the variability within and across episodic memory paradigms.

## Figures and Tables

**Fig. 9:**
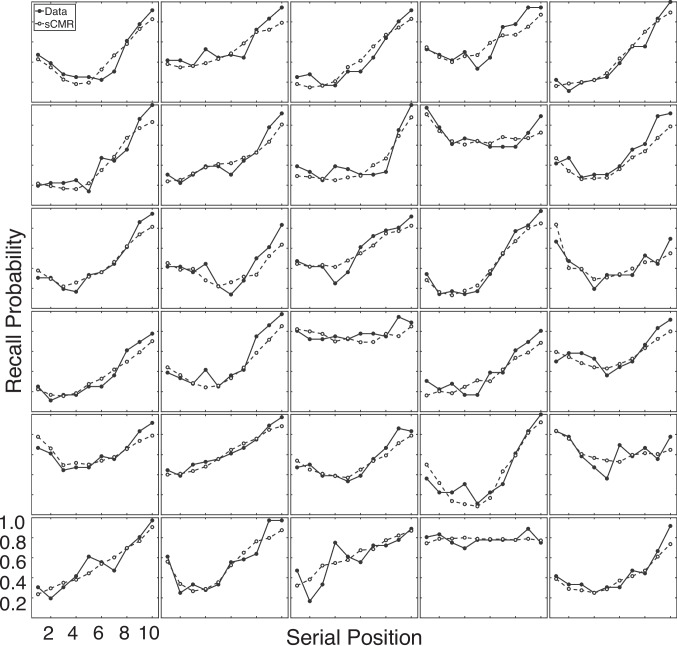
sCMR predictions and experimental data of serial position curves by participant in free recall. Ascending participant numbers go from left to right in each row, and then down each column. Data are from Golomb et al. (2008)

**Fig. 10:**
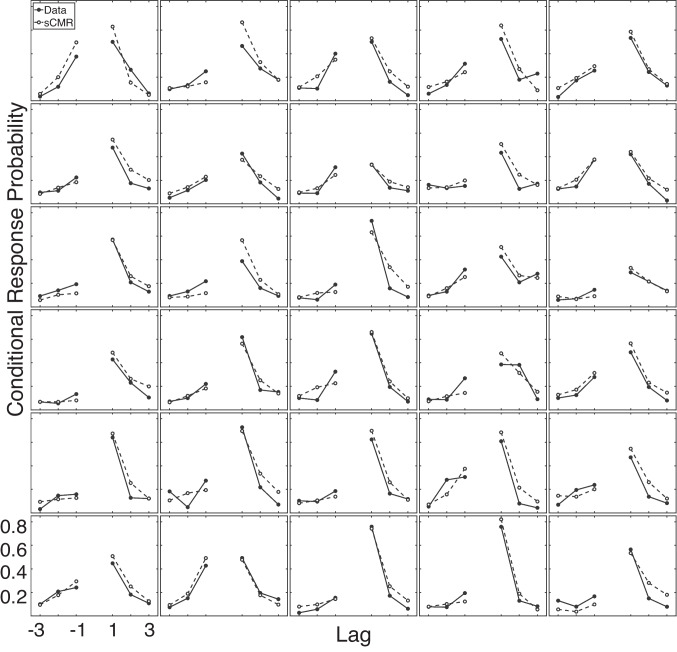
sCMR predictions and experimental data of conditional response probability as a function of lag across all valid output positions in free recall. Ascending participant numbers go from left to right in each row, and then down each column. Data are from Golomb et al. (2008)

**Fig. 11:**
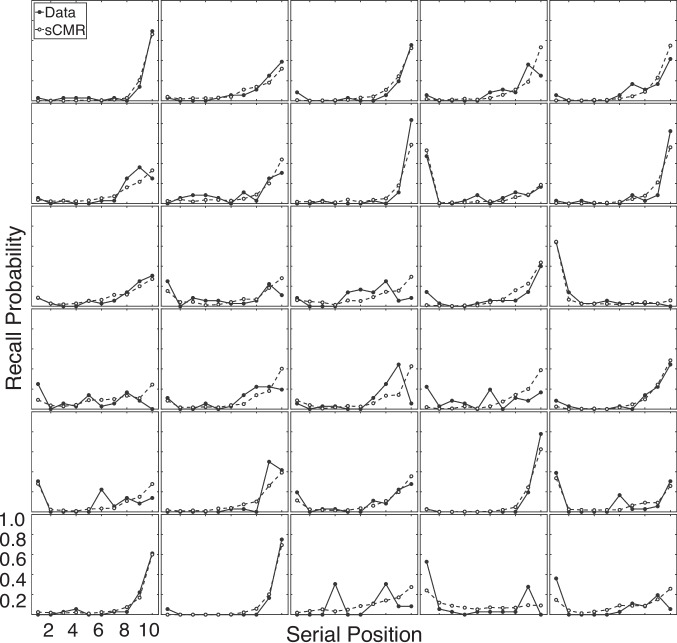
sCMR predictions and experimental data of probability of first recall by participant in free recall. Ascending participant numbers go from left to right in each row, and then down each column. Data are from Golomb et al. (2008)

**Fig. 12:**
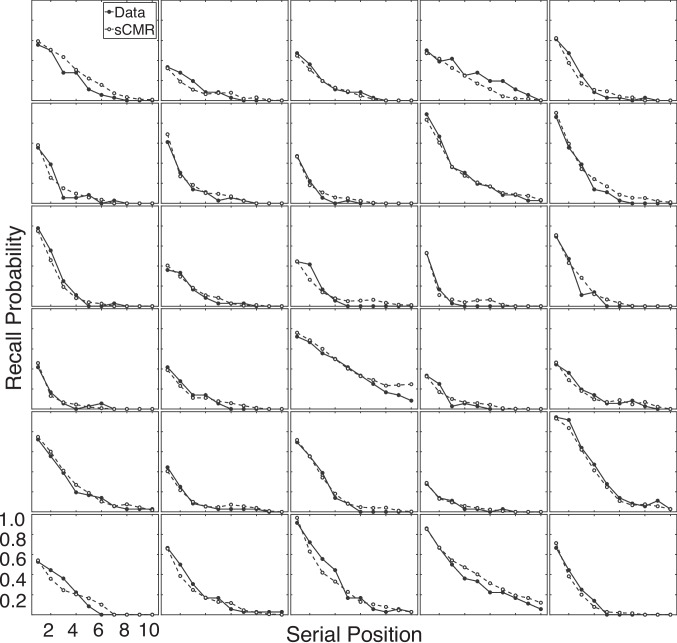
sCMR predictions and experimental data of serial position curves by participant in serial recall. Ascending participant numbers go from left to right in each row, and then down each column. Data are from Golomb et al. (2008)

**Fig. 13:**
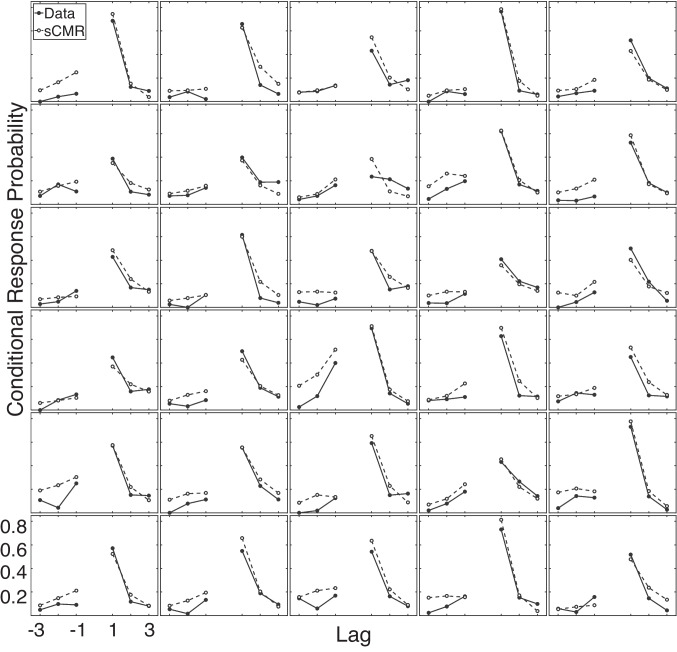
sCMR predictions and experimental data of conditional response probability as a function of lag across all valid output positions in serial recall. Ascending participant numbers go from left to right in each row, and then down each column. Data are from Golomb et al. (2008)

**Fig. 14:**
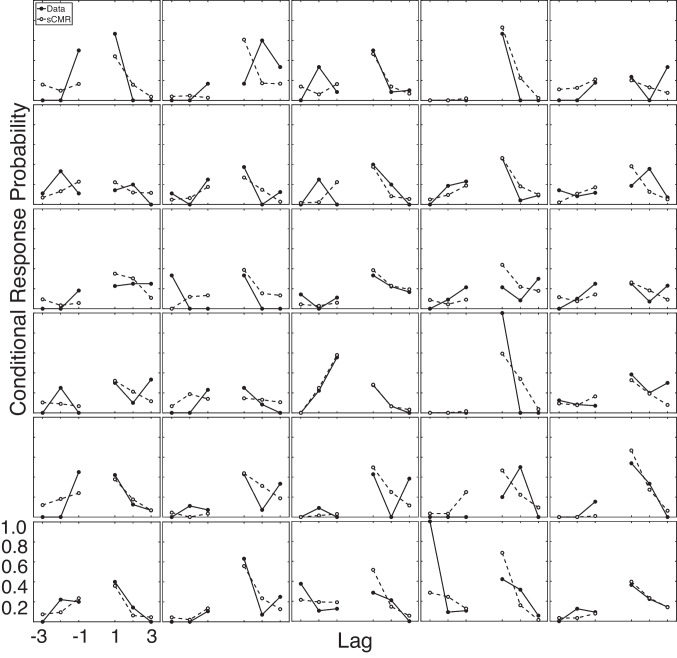
sCMR predictions and experimental data of conditional response probability as a function of lag following the first-order error in serial recall. Ascending participant numbers go from left to right in each row, and then down each column. Note the change in the *y*-axis to accommodate two participants with a conditional response probability value of one. Data are from Golomb et al. (2008)

**Fig. 15:**
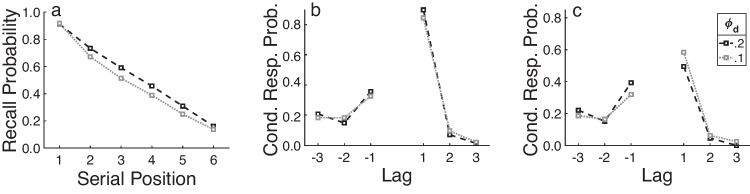
sCMR predictions and experimental data of the primacy, recency, and fill-in effects with a very low value of $\phi _d$. In serial recall, lower values of this parameter can produce less of a primacy effect in the serial position curve and more forward asymmetry in the lag-CRP. See text for details. **a** Serial position curves. **b** Conditional response probability as a function of lag. **c** Conditional response probability as a function of lag following the first-order error. Data are simulated based on Osth and Dennis (2015a; b)

## Data Availability

Computer code used to generate the present simulations is available at the Open Science Foundation repository: https://osf.io/qsr7p/. This code relies on analysis software available from https://github.com/vucml/EMBAM, as well as data sets available from http://memory.psych.upenn.edu/Data Archive#2008 (Simulation Set 1) and https://osf.io/8zycm/ (Simulation Set 2).

## Appendix

### Simulation Set 1 by Participant

sCMR was fit to each participant individually, with a single set of parameters for each participant (see the *Method* subsection for *Simulation Set 1*). Whereas Figs. 2 a, b, c, d, e, and f provide the average experimental data and average sCMR fits across participants, the individual model fits for each participant are provided, respectively, in Figs. 9, 10, 11, 12, 13, and 14.

### Influence of Primacy Decay Parameter in Serial Recall

In sCMR, the $\phi _d$ parameter controls the rate of decay of the extra strength attributed to primacy items. However, at very low values, this can actually decrease recall of primacy items. As a further demonstration of this intuition, Fig. 15 shows sCMR’s predictions, once again fit to the middle third of participants using the best-fit parameters for Simulation 2a in Table 1 except $\phi _d$ is half of its best-fit value (0.101). Overall recall is even lower and decreases more rapidly with serial position than the higher best-fit value for this participant group (0.202). This reflects the competition between items preceding and following the first-order error. Indeed, now the lag-CRP following the first-order error exhibits the in-fill effect. This suggests that, in sCMR, an increased primacy advantage for early list items produces more competition between which item will be recalled next. Thus, in addition to the preceding item, the following item also serves as a strong competitor and is more likely to be recalled.

## Footnotes

1 Currently, sCMR can only recall items studied in the list, but if a participant begins with an error then the probabilities across all valid serial positions will not sum to one. Thus, in the probability of first recall functions provided to sCMR, recall of all serial positions aside from the first item was normalized so that the values at all serial positions had a sum of one. Recall of the first item, the most likely possibility, was consistent with the value in the behavioral data, and the normalization was applied to the remaining serial positions. This also prevented sCMR from (the rare empirical occurrence of) recalling an intrusion or not recalling an item at all in the first serial position.

2 From the original study of 36 participants, I excluded five participants who performed free recall in their serial recall session—beginning recall with the first item for less than 25% of trials—and excluded one participant who performed serial recall in the free recall session, as that participant initiated recall with the first two list items for over 50% of trials. This more conservative criterion was meant to ensure that a failure of sCMR to capture serial or free recall data was not simply due to qualitatively atypical recall patterns.
